# Comparative plastome analysis of the sister genera *Ceratocephala* and *Myosurus* (Ranunculaceae) reveals signals of adaptive evolution to arid and aquatic environments

**DOI:** 10.1186/s12870-024-04891-2

**Published:** 2024-03-20

**Authors:** Jing Long, Wen-Chuang He, Huan-Wen Peng, Andrey S. Erst, Wei Wang, Kun-Li Xiang

**Affiliations:** 1 State Key Laboratory of Plant Diversity and Prominent Crops, Institute of Botany, Chinese Academy of Sciences, Beijing, 100093 China; 2 University of Chinese Academy of Sciences, Beijing, 100049 China; 3 China National Botanical Garden, Beijing, 100093 China; 4 Shenzhen Branch, Guangdong Laboratory of Lingnan Modern Agriculture, Genome Analysis Laboratory of the Ministry of Agriculture and Rural Affairs, Agricultural Genomics Institute at Shenzhen, Chinese Academy of Agricultural Sciences, Shenzhen, 518120 China; 5 Central Siberian Botanical Garden, Siberian Branch of Russian Academy of Sciences, Zolotodolinskaya Str. 101, Novosibirsk, 630090 Russia

**Keywords:** Adaptive evolution, Phylogenomics, Plastome, Positive selection, Ranunculaceae, Structural variation

## Abstract

**Background:**

Expansion and contraction of inverted repeats can cause considerable variation of plastid genomes (plastomes) in angiosperms. However, little is known about whether structural variations of plastomes are associated with adaptation to or occupancy of new environments. Moreover, adaptive evolution of angiosperm plastid genes remains poorly understood. Here, we sequenced the complete plastomes for four species of xerophytic *Ceratocephala* and hydrophytic *Myosurus*, as well as *Ficaria verna*. By an integration of phylogenomic, comparative genomic, and selection pressure analyses, we investigated evolutionary patterns of plastomes in Ranunculeae and their relationships with adaptation to dry and aquatic habitats.

**Results:**

Owing to the significant contraction of the boundary of IR_A_/LSC towards the IR_A_, plastome sizes and IR lengths of *Myosurus* and *Ceratocephala* are smaller within Ranunculeae. Compared to other Ranunculeae, the *Myosurus* plastome lost *clpP* and *rps16*, one copy of *rpl2* and *rpl23*, and one intron of *rpoC1* and *rpl16*, and the *Ceratocephala* plastome added an *infA* gene and lost one copy of *rpl2* and two introns of *clpP*. A total of 11 plastid genes (14%) showed positive selection, two genes common to *Myosurus* and *Ceratocephala*, seven in *Ceratocephala* only, and two in *Myosurus* only. Four genes showed strong signals of episodic positive selection. The *rps7* gene of *Ceratocephala* and the *rpl32* and *ycf4* genes of *Myosurus* showed an increase in the rate of variation close to 3.3 Ma.

**Conclusions:**

The plastomic structure variations as well as the positive selection of two plastid genes might be related to the colonization of new environments by the common ancestor of *Ceratocephala* and *Myosurus*. The seven and two genes under positive selection might be related to the adaptation to dry and aquatic habitats in *Ceratocephala* and *Myosurus*, respectively. Moreover, intensified aridity and frequent sea-level fluctuations, as well as global cooling, might have favored an increased rate of change in some genes at about 3.3 Ma, associated with adaptation to dry and aquatic environments, respectively. These findings suggest that changing environments might have influenced structural variations of plastomes and fixed new mutations arising on some plastid genes owing to adaptation to specific habitats.

**Supplementary Information:**

The online version contains supplementary material available at 10.1186/s12870-024-04891-2.

## Background

The plastid is an organelle with an essential role in the photosynthesis of green plants [1]. Typically, angiosperm plastid genomes (plastomes) display a quadripartite circular structure with sequences ranging from 130 to 170 kb in length [2]. The complete plastome consists of one large single copy (LSC) and one small single copy (SSC) regions, flanked by two inverted repeats (IR_A_ and IR_B_) [2, 3]. Generally, the plastomes of angiosperms are highly conserved in terms of gene content and structure, but there is considerable variation resulting from the expansion and contraction of IRs [4], the addition, loss and pseudogenization of genes [5], the inversion of genes and regions [6], and polymorphic simple sequence repeats (SSRs) [7]. Some studies have indicated that plastomic variations of heterotrophic plants (parasites or mycoheterotrophs) are correlated with their unique lifestyle [8]. For example, mycoheterotrophic Neottieae (Orchidaceae) have lost plastid NADH dehydrogenase-like complex and photosynthesis-related genes [9]. To date, we know little about whether plastomic variations of an angiosperm group are associated with adaptation or occupancy of new environments, such as dry and aquatic habitats.

Protein-coding genes of a complete angiosperm plastome can be divided into five functional groups, i.e., plastid NADH dehydrogenase-like complex, photosynthesis-related, plastid-encoded RNA polymerase, plastid ATP synthase, and housekeeping [8, 9]. Some plastid protein-coding genes have been reported under positive selection in different taxa, such as seven NADH dehydrogenase-like complex genes (*ndhA*, *ndhB*, *ndhE*, *ndhD*, *ndhF*, *ndhG*, and *ndhK*) in *Allium* (Amaryllidaceae) [10], four phyotosynthesis-related genes (*psaA*, *psbB*, *psbD*, and *psbH*) in *Oryza* (Poaceae) [11], one plastid-encoded RNA polymerase gene (*rpoC2*) in *Cardamine resedifolia* (Brassicaceae) [12], three ATP synthase genes (*atpA*, *atpB*, and *atpI*) in Dipsacales [13], and three housekeeping genes (*clpP*, *ycf2* and *rps14*) in *Euphrasia* (Orobanchaceae) [14]. The identification of positively selected genes has been a powerful tool for investigating organismal adaptation to climate changes [10, 15, 16]. In particular, identifying episodic positive selection can be used to determine when adaptation to a specifically environmental condition occurred. As an example, Zecca et al. [17] show that the *psbK*, *rpl20*, *rpoB*, and *rps11* genes in Vitaceae had episodic signatures of positive selection and experienced an increase in the rate of variation close to the Cretaceous–Palaeogene transition, which might be influenced by intense environmental perturbations during the transition. It is well-known that extant angiosperms occupy various environments. To gain a better understanding of adaptive evolution of angiosperm plastid genes to environmental changes, we need to examine more taxa growing in diverse environments in a phylogenetic context.

The angiosperm family Ranunculaceae, well known as the buttercup family, is an important herbaceous element of mountain ecosystems in the Northern Hemisphere [18]. Ranunculeae is the largest tribe in this family and consists of 19 genera with approximately 650 species [19, 20]. Within Ranunculeae, most of genera grow in mesophytic habitats except *Ceratocephala*, *Myosurus* and some species of *Ranunculus* [19]. *Ceratocephala* contains three or four species that are mainly distributed in dry regions of Central Asia with one in New Zealand, and *Myosurus* has about fifteen species that inhabit wet or seasonally wet regions of all continents. Phylogenetic analyses support the sister relationship between xerophytic *Ceratocephala* and hydrophytic *Myosurus* [20–22]. Molecular clock estimates suggest a stem age of 31–42 Ma for the *Ceratocephala*-*Myosurus* clade, and the split time of these two genera as 25–35 Ma [22]. From the late Eocene onwards, global climate has been altered dramatically [23]. Hence, *Ceratocephala* and *Myosurus* provide a good opportunity to explore evolutionary adaptation of plastomes to arid and aquatic environments.

In this study, we first sequenced the complete plastomes for two species of *Ceratocephala* and two species of *Myosurus*, as well as one of *Ficaria verna*. Then, we performed phylogenetic analyses and estimated divergence times for Ranunculeae in combination with the plastomic data of seven other species of Ranunculeae. Within the dated phylogenetic framework, we finally inferred the patterns of structural variations of the plastomes, identified genes that are under positive selection, and explored the changes through time in the rates of variation of plastid genes of *Ceratocephala* and *Myosurus* under episodic positive selection. These analyses will contribute to a better understanding of how angiosperm plastomes have evolved and adapted to arid and aquatic environments through large-scale environmental changes.

## Results

### Genome feature

**Fig. 1:**
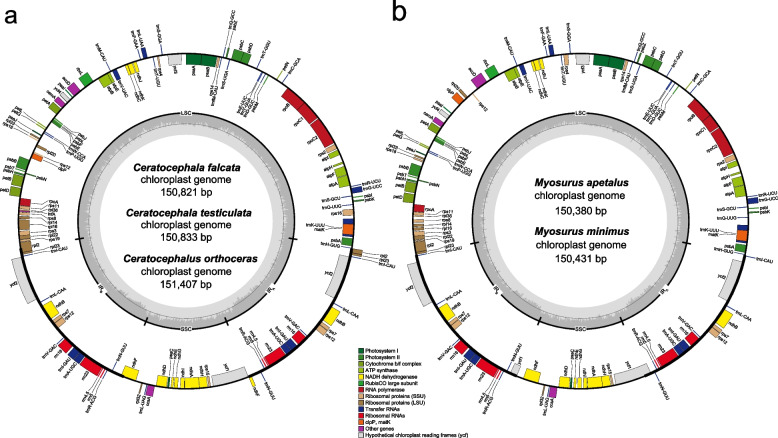
Plastomes of *Ceratocephalus* (**a**) and *Myosurus* (**b**). The genes inside and outside of the circle are transcribed in clockwise and counterclockwise directions, respectively. Genes belonging to different functional groups are shown in different colors. The thick lines indicate the extent of IR_A_ and IR_B_ that separate the genomes into SSC and LSC regions. IR, inverted repeats; SSC, small single-copy; LSC, large single-copy

The complete plastomes of five species of Ranunculeae were de novo assembled. Combining them with seven previously published plastomes (Table S1), we performed comparative analysis of 12 Ranunculeae plastomes with a genome size ranging from 150.4 kb in *Myosurus* to 157.3 kb in *Halerpestes* (Figs. 1, S1, S2; Table S1). All plastomes exhibited the typical angiosperm quadripartite structure, which comprises a LSC (83.6–86.7 kb) and a SSC (18.4–22.0 kb) region, separated by two IR regions (23.3–25.8 kb). The plastome sizes of *Ceratocephala* (150.8–151.4 kb) and *Myosurus* (*ca.*150.4 kb) were smaller than that of other Ranunculeae genera (154.2–157.3 kb). The GC content of the plastomes in *Ceratocephala* (38.4%) and *Myosurus* (39.8%) was higher than that of other Ranunculeae genera, similar trends in GC content variations were found in the LSC, SSC, and IR regions in Ranunculeae.

We performed high-quality gene annotation for the 12 plastomes, and identified 76–79 plastid protein-coding genes, 29–30 tRNA genes, and 4 rRNA genes (Table S1). Within Ranunculeae, *Ceratocephala* contains the highest number of plastid protein-coding genes (79), and *Myosurus* has the lowest number of plastid protein-coding genes (76). The GC3s value of protein-coding genes in *Myosurus* and *Ceratocephala* was generally higher than that of other Ranunculeae genera (Fig. S3).

### Phylogenetic analysis and divergence time estimation

**Fig. 2:**
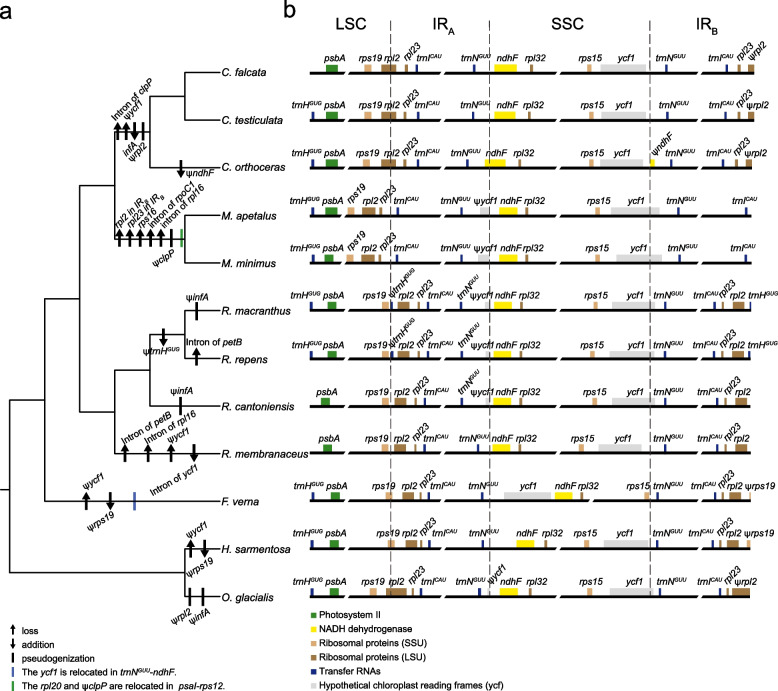
Comparison of plastomic structures in Ranunculeae. **a** Scenarios of gene losses, additions, pseudogenizations, and relocations along the phylogenomic tree of Ranunculeae. The tree was generated based on the 78 protein-coding plastid genes (see Fig. S4 for the whole tree). **b** Variations of the LSC, IRs, and SSC region boundaries (see details in Fig. S5). The ψ-symbol shows putative pseudogenes. IR, inverted repeats; SSC, small single-copy; LSC, large single-copy

Phylogenetic relationships within Ranunculeae were reconstructed based on the 78 plastid protein-coding gene data (Figs. 2a and S4). *Halerpestes* and *Oxygraphis* formed a clade, sister to the other Ranunculeae. *Ceratocephala* and *Myosurus* formed a clade with strong support (bootstrap support (BS) = 100%), sister to *Ranunculus*. The monophyly of each of *Ceratocephala*, *Myosurus* and *Ranunculus* is strongly supported.

**Fig. 3:**
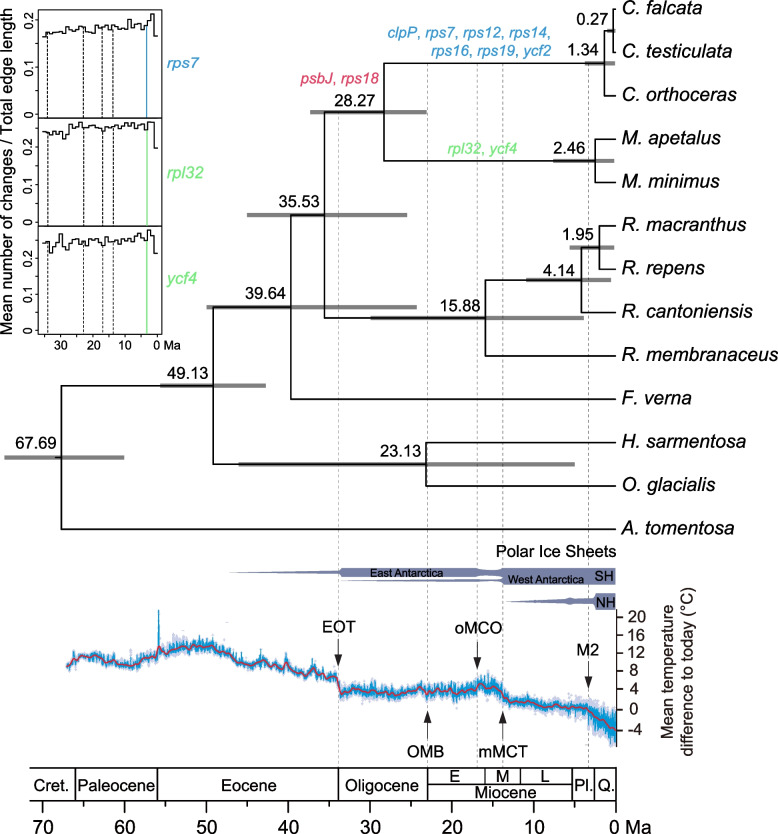
Combined chronogram and positive selection analyses of Ranunculeae. Dating analysis was performed using BEAST based on the 78 plastid protein-coding gene data. The grey bars represent 95% highest posterior density intervals. The 11 genes identified under positive selection are shown in the tree. The depiction of temperature changes is modified from Westerhold et al. [23]. The dashed lines represent the five periods investigated in this study: 34, 23.03, 17, 13.9, and 3.3 Ma; and the colored line indicates the period when the rate of variation significantly increased. Genes positively selected in *Ceratocephala* and *Myosurus* are in red; genes positively selected in *Ceratocephala* are in blue; genes positively selected in *Myosurus* are in green. Changes through time plots generated from sampled stochastic character maps for the three genes found to be under episodic positive selection is present in the upper left (see Fig. S7 and Table S8 for details). SH, Southern Hemisphere; NH, Northern Hemisphere; EOT, Eocene–Oligocene Transition; OMB, Oligogocene-Miocene boundary; oMCO, onset of Miocene Climate Optimum; mMCT, middle Miocene Climate Transition; M2, first major glacial event in the NH; Cret., Cretaceous; M., Middle; P., Pliocene; Q., Quaternary

Divergence time estimates for Ranunculeae are shown in Fig. 3. The stem and crown group ages of Ranunculeae are 67.69 Ma (95% highest posterior density (HPD): 60.01–74.64) and 49.13 Ma (95% HPD: 42.71–55.6), respectively. The *Ceratocephala*-*Myosurus* clade originated at 35.53 Ma (95% HPD: 25.44–44.99). The split of *Ceratocephala* and *Myosurus* occurred at 28.27 Ma (95% HPD: 23.08–37.28)*. Ceratocephala* and *Myosurus* began to diversify at 1.34 Ma (95% HPD: 0.06–3.72) and 2.46 Ma (95% HPD: 0.15–7.58), respectively.

### Plastome structural variations

To assess the plastomic synteny among the Ranunculeae species distributed in different habitats, structural variations of Ranunculeae plastomes were investigated (Figs. 2 and S5; Table S2). A total of 88,069 nucleotide variations were detected in all sampled Ranunculeae species, in which the majority of variants (92.85%) were found in non-coding regions. For the coding regions, 38.94% of the nucleotide variants were functionally missense variations distributed in 56 protein-coding genes. Extensive functional variations were identified among the plastomes of *Ceratocephala*, *Myosurus*, and *Ranunculus*, including 3,156 missense variants in 71 genes between *Ceratocephala* and *Myosurus*, 2,533 missense variants in 71 genes between *Ceratocephala* and *Ranunculus*, and 3,351 missense variants in 70 genes between *Myosurus* and *Ranunculus* (Table S2). Accordingly, these three genera possessed distinct plastid gene components, i.e., the *Ceratocephala* plastome added an *infA* and lost two introns of *clpP*, the *Myosurus* plastome lost *clpP*, *infA*, *rps16*, one copy of *rpl2* and *rpl23*, and one intron of *rpl16* and *rpoC1*, and the *Ranunculus* plastomes presents a more diverse variations in different species (Fig. 2a; Table S3).

To investigate the contraction and expansion events of the Ranunculeae plastomes, we compared the exact borders of the IR/SC regions (Figs. 2 and S5; Table S1). For the LSC/IR_A_ region, *rpl2* spanned the region while a pseudogene fragment, namely ψ*rpl2* was located at the IR_B_ region in *Ceratocephala* with 590 bp and in *Oxygraphis* with 1,036 bp; *rps19* spanned the same region while a pseudogene fragment (ψ*rps19*) was located at the IR_B_ region in *Ficaria* with 78 bp and *Halerpestes* with 174 bp. For the SSC/IR_B_ region, *ycf1* spanned the region while a pseudogene fragment (ψ*ycf1*) was located at the IR_A_ region with a length range of 57–988 bp in *Myosurus*, *Oxygraphis*, and three species of *Ranunculus* (i.e., *R. cantoniensis*, *R. macranthus*, and *R. repens*)*.* In the *Ficaria* plastome, *ycf1* was located in *trnN*^*GUU*^-*ndhF* at the SSC region, which was unique in the Ranunculeae. Meanwhile, ψ*ycf1* was lost in the plastomes of *Ficaria*, *Halerpestes*, and *Ceratocephala*. For the LSC/IR_B_ region, *trnH*^*GUG*^ spanned the region while a pseudogene fragment (ψ*trnH*^*GUG*^) was located at the IR_A_ region with 23 and 61 bp in *R. cantoniensis* and *R. repens*, respectively. Additionally, in the LSC region, there was a difference among *Myosurus* and other genera, the former having the ψ*clpP* and *rpl20* relocated to the region of *psaI-rps12* (Fig. 1).

### Selection pressure analysis

To determine potential genes that could show signals of adaptive evolution to different habitats, we used the likelihood ratio test to search the optimal model in selection pressure analysis. For all 78 plastid protein-coding genes, the free-ratio model (m2), assuming three independent *ω* (the ratio of *d*_*N*_/*d*_*S*_), was identified as best-fit to *Ceratocephala*, *Myosurus*, and other Ranunculeae (Tables S4–S6). In the *Ceratocephala* plastome, we identified nine positively selected genes (*ω* > 1), including a photosystem II factor gene (*psbJ*), a protein-modifying enzyme gene (*clpP*), six ribosome small subunit genes (*rps7*, *rps12*, *rps14*, *rps16*, *rps18*, and *rps19*), and an unknown function gene (*ycf2*). In the *Myosurus* plastome, four genes showed signatures of positive selection (*ω* > 1), including a photosystem II factor gene (*psbJ*), a ribosome small subunit gene (*rps18*), a ribosome large subunit gene (*rpl32*) and a photosystem assembly factor gene (*ycf4*).

### Investigating trait changes through time

To determine the period when the gene underwent positive selection, we coded the different amino acid sites as character states for *Ceratocephala*, *Myosurus*, and *Ranunculus* (Fig. S6; Table S7). All eleven genes under positive selection fitted the all-rates-different (ARD) likelihood model for discrete character evolution. Changes through time plots generated from sampled stochastic character maps for each gene are shown in Fig. S7.

As obviously temperature and/or precipitation changes occurred at 34 Ma, 23.03 Ma, 17 Ma, 13.9 Ma, and 3.3 Ma, we chose these five periods to generate and fit models in which the rate of change of the composite trait varied between different times on the reference chronogram (Fig. 3; Table S8). By comparing the equal-rates (ER), the symmetrical (SYM), and ARD likelihood models at five periods, we selected the periods in which the rate variation of genes was most likely to change. The ARD model was identified as best-fit for all genes except *clpP* and *rps16* (for which no best-fit model was identified); and the *rpl32*, *rps7*, *rps18*, and *ycf4* genes showed strong signals of episodic positive selection (*P* < 0.05; Table S8). The changes through time plots indicate that the *rpl32*, *rps7*, and *ycf4* genes showed an increase in the rate of variation at about 3.3 Ma, whereas the increases of rate of the *rps18* gene did not occur in any of the five periods (Fig. 3; Fig. S7).

## Discussion

### Structural variations of plastomes in *Ceratocephala* and *Myosurus*

Our results show that *Ceratocephala* and *Myosurus* formed a clade, sister to *Ranunculus* (Figs. 2a and S4), consistent with previous studies [21, 22, 24]. Within Ranunculeae, the plastome size of *Myosurus* is the smallest, followed by *Ceratocephala*. Similarly, the IR regions of *Myosurus* and *Ceratocephala* are shorter than that of other Ranunculeae (Table S1). The *rpl2* gene is located in the LSC/IR_A_ region in *Ceratocephala* and in the LSC region in *Myosurus*, respectively, whereas in other Ranunculeae genera, this gene is located in the IR region. In most angiosperms, the *rpl2* gene is located in the IR region, such as in *Bletilla* (Orchidaceae) [25], *Piper* (Piperaceae) [26], and *Mahonia* (Berberidaceae) [27]. Thus, the contraction of the IR_A_ towards the LSC resulted in the smaller plastome size and shorter IR length of *Ceratocephala* and *Myosurus* (Fig. 2b).

The expansion or contraction of the IR-LSC boundary regions is considered as a primary mechanism causing the length variation of angiosperm plastomes [28]. The expansions or contractions of the IRs into or out of adjacent single-copy regions are frequently observed in angiosperm plastomes [29], which may be related to the colonization of particular habitats. The species of *Corydalis* (Papaveraceae) occur from sea level to more than 6,000 m altitude, often in forest understoreys, alpine meadows and screes, deserts, and steppes, and the expansion of IR region were reported widely in this genus [30]. Among the four species of *Echinacanthus* (Acanthaceae), the one distributed in the western Himalaya has the plastome with the contraction of the IR region, compared to the other three restricted to the Sino-Vietnamese karst region [31]. Thus, the contraction of IR regions might have occurred in the most recent common ancestor (MRCA) of *Ceratocephala* and *Myosurus*, in association with the colonization of new habitats and the divergence from the mesophytic Ranunculeae.

Although the IR regions became smaller in *Ceratocephala* and *Myosurus*, their GC average contents are higher than in other Ranunculeae genera (Table S1). We also find that the GC3s values of 78 protein-coding genes in *Ceratocephala* and *Myosurus* were higher than that of other Ranunculeae genera (Fig. S3). Generally, high GC content imparts more stability to the genome [32]. Compared to mesophytic Ranunculeae, the plastome of xerophytic *Ceratocephala* has a higher GC content. Genomic DNA with high GC content is considered to be more thermostable in *Welwitschia*, a unique desert xerophytic genus [33]. The plastome of hydrophytic *Myosurus* also has a relatively higher GC content than that of mesophytic Ranunculeae. Thus, the higher GC content may have facilitated *Ceratocephala* and *Myosurus* to adapt to new environments, such as dry and aquatic habitats. Similarly, Jabbari and Bernardi [34] have suggested that the larger amount of GC base pairs in the genome of vertebrates has an important role in their adaptations to various environments.

*Ceratocephala* and *Myosurus* are sister to each other, and contain 79 and 76 plastid protein-coding genes, respectively (Table S1). Compared to other Ranunculeae genera, *Ceratocephala* added one *infA* and lost two introns of *clpP* and ψ*ycf1*, whereas in the *Myosurus* plastome, *rps16* was lacking, *rpoC1* and *rpl16* each lost an intron, and *clpP* was changed into ψ*clpP* (Fig. 2; Table S3). Variations of plastomic gene contents, including loss, addition, and pseudogenization are often related to environmental selection pressure. For example, in the plastome of *Azadirachta indica* (Meliaceae), the ten *ndh* genes (*ndhA*, *ndhC*, *ndhD*, *ndhE*, *ndhF*, *ndhG*, *ndhH*, *ndhI*, *ndhJ*, and *ndhK*) were lost and *ndhB* became a pseudogene, which might be correlated with holoparasitic habit and lower light habitats [35]. Whether the variations of plastomic gene contents in *Ceratocephala* and *Myosurus* are associated with their respective dry and aquatic habitats need to be studied in the future.

### Plastomic genes under positive selection in *Ceratocephala* and *Myosurus*

A total of eleven genes are under positive selection in the plastomes of *Ceratocephala* and *Myosurus* (Fig. 3)*.* Among them, the *psbJ* and *rps18* genes are positively selected both in *Ceratocephala* and *Myosurus*, suggesting that these two genes might have been related to the colonization of new environments by the MRCA of these two genera, and thereby to the divergence from their mesophytic ancestor. In particular, the seven genes (*clpP*, *rps7*, *rps12*, *rps14*, *rps16*, *rps19*, and *ycf2*) are only found to have signals of positive selection in the *Ceratocephala* plastome, implying that these seven genes might have been associated with the adaptation of *Ceratocephala* to arid environments. Zhong et al. [36] also found that the *ycf2* was gene under positive selection in *Helianthus tuberosus* (Asteraceae), which grows in saline, alkaline and dry conditions as a widely cultivated plant in Northwest China. The two genes (*rpl32* and *ycf4*) are only found to have signals of positive selection in the *Myosurus* plastome, implying that they might have been associated with the adaptation of *Myosurus* to aquatic environments. In *Nicotiana tabacum* (Solanaceae), the *ycf4* gene has been document to be essential for transcriptional gene regulation and plant photoautotrophic growth [37].

Our divergence time estimation shows that *Ceratocephala* and *Myosurus* originated in the late Eocene (35.53 Ma, 95% HPD: 25.44–44.99) and diverged in the late Oligocene (28.27 Ma, 95% HPD: 23.08–37.28; Fig. 3). Since the late Eocene, dramatic changes in global temperature and/or precipitation occurred at least during five periods (Fig. 3) [23, 38]: the Eocene–Oligocene Transition (EOT; *ca.* 34 Ma) [39], the Oligocene–Miocene boundary (OMB; *ca. *23.03 Ma) [40], the onset of the Miocene Climatic Optimum (oMCO; *ca.* 17 Ma) [41], the mid Miocene Climate Transition (mMCT; *ca.* 13.9 Ma) [42], and the first major glacial event in the Northern Hemisphere (M2; *ca.* 3.3 Ma) [23]. Our results indicate that among the eleven genes under positive selection, the four genes (*rpl32*, *rps7*, *rps18*, and *ycf4*) showed strong signals of episodic positive selection (*P* < 0.05; Table S8). Importantly, we identified the three genes (*rpl32*, *rps7*, and *ycf4*) showing an increase in the rate of variation at about 3.3 Ma, a timing coinciding with one of the five periods (Fig. 3). These three genes belong to housekeeping genes: *rpl32* is a ribosome large subunit gene, *rps7* is a ribosome small subunit gene, and *ycf4* is a photosystem assembly factor.

The change through time plot indicates that for *Ceratocephala*, the rate of variation of the *rps7* gene increased around 3.3 Ma (Fig. 3), a timing when the first major glacial event in the Northern Hemisphere occurred and accordingly global temperature dropped drastically [23]. During this period, the northeastern and southeastern Tibet Plateau experienced a rapid uplift and outward growth [43, 44]. These events might have resulted in the increasing aridification in central Eurasia. Paleoclimate modeling indicates that the precipitation markedly decreased at 4 Ma [38]. Moreover, the Central Asian arid region was beyond the scope of the Asian monsoon precipitation [45]. Thus, the cooling and aridification in Central Asia during the M2 might have promoted the *rps7* gene to arise new favorable mutations in xerophytic and annual *Ceratocephala*, which could further be fixed by natural selection in arid environments.

For *Myosurus*, both the *rpl32* and *ycf4* genes showed an increase in the rate of variation also probably at about 3.3 Ma (Fig. 3). In the last 5 Ma, highly frequent oscillations in sea-level occurred [46], including at least 58 rapid rises over 40 m [47, 48]. Frequent sea-level fluctuations, as well as global cooling (Fig. 3), might have led to annual *Myosurus* to adapt to changing aquatic habitats. Thus, the *rpl32* and *ycf4* genes could have evolved rapidly in the Pliocene and further fixed favorable mutations, possibly in association with the adaptation to wet environments.

## Conclusions

In this study, we de novo assembled the complete plastome sequences of five species from *Ceratocephala*, *Ficaria*, and *Myosurus*, and updated the annotation of plastomes for seven other Ranunculeae species. Plastid phylogenomic analysis strongly supports a sister relationship between xerophytic *Ceratocephala* and hydrophytic *Myosurus*. We discover that the plastome sizes of *Ceratocephala* and *Myosurus* are smaller than that of other mesophytic Ranunculeae, mainly due to the contraction of the IR region. The addition, loss and pseudogenization of plastid genes were found in these two genera. Eleven plastid genes showed positive selection in *Ceratocephala* and *Myosurus*. Among them, four genes showed strong signals of episodic positive selection. Importantly, the rate of variation of the *rps7* gene in *Ceratocephala* increased around 3.3 Ma, possibly associated with the adaptation to dry habitats owing to the cooling and aridification in Central Asia; the *rpl32* and *ycf4* genes in *Myosurus* showed an increase in the rate of variation at about 3.3 Ma, possibly associated with the adaptation to aquatic habitats resulting from sea-level fluctuations and global cooling. Our integration of phylogenomic, comparative genomic, and selection pressure analyses can be used to explore adaptive evolution of plastomes of other plant groups to specific habitats.

## Materials and methods

### Sample sequencing, data assembly and annotation

We sampled three species of *Ceratocephala* and two of *Myosurus*. The other three mesophytic genera (*Ficaria*, *Halerpestes*, *Oxygraphis*) and four mesophytic species of *Ranunculus* in Ranunculeae were also included (Table S1). Based on the results of previous studies [22, 24], we selected *Anemone tomentosa* of Anemoneae, sister to Ranunculeae, as outgroup. The samples of *Ceratocephala orthoceras*, *C. testiculata*, *Myosurus apetalus*, *M. minimus*, and *F. verna* were newly collected from Altay (Xinjiang, China), Huocheng (Xinjiang, China), Nevada (USA), Malkow (Poland), Wieliczka (Poland), respectively, and were deposited in Herbarium, Institute of Botany, Chinese Academy of Sciences, Beijing (PE). Their formal identification was undertaken by Wei Wang and Andrey S. Erst. No specific permissions or licenses were required for our collections and experiments.

Genomic DNA of the five species was extracted from silica gel-dried leaves or herbarium specimens and purified using the Tiangen Isolation/Extraction/Purification Kit (Tiangen Biotech (Beijing) Co., Ltd.). Short insert of 300–500 bp libraries were prepared for sequencing on the Illumina HiSeq X-Ten platform.

The plastome was de novo assembled using GetOrganelle v1.7.6.1 [49] and was annotated by PGA [50] with the plastomes of *Ceratocephala*, *Halerpestes*, *Oxygraphis*, and *R. macranthus* as references. We used OGDRAW v1.3.1 [51] to visualize the circular plastome map with subsequent manual editing. The amino acid sequences of 78 plastid protein-coding gene regions were extracted and each was aligned in MAFFT v7 [52]. DNA sequences were then aligned using PAL2NAL v14 [53]. We also updated the annotation of plastomes for the other seven species sampled in the study. We used the program DNAsp v6.12.03 [54] to analyze the synonymous codon usage of 78 protein-coding genes by calculating the values of GC3s.

### Phylogenetic analyses and divergence time estimation

The maximum likelihood (ML) analysis was performed using RAxML v8.2.12 [55] with 1,000 replicates under the GTRGAMMA model. The analysis was carried out based on the concatenation of coding regions of 78 protein-coding genes.

Divergence times were estimated in BEAST v2.1.3 [56]. Fossil achenes of *Myosurus* sp. were found from the Oligocene [57]. We set a 23.03 Ma constraint for the split between *Ceratocephala* and *Myosurus*, with a lognormal distribution. The offset (minimum age constraint) was set to be equal to the age of the fossil. The 95% upper bound of the distribution (soft maximum) was set by adjusting the standard deviation with 1.25. We also used two calibration points, taking ages estimated in the recent broader study of Ranunculeae [22]: 49.4 Ma (95% HPD: 42.71–55.60) for the crown group age of Ranunculeae and 68.95 Ma (95% HPD: 60.01–74.64) for the root age. Prior normal distributions were assigned for these two calibrations with standard deviations of 3.5 and 3.7, respectively. We used an uncorrelated lognormal relaxed clock model of rate variation across branches, a Yule prior, and the GTR model for each gene partition separately.

Parameters were estimated using four independent runs of 100,000,000 Markov chain Monte Carlo (MCMC) generations each, with sampling every 2,000 generations. Convergence was evaluated in Tracer v1.7.1 [58]. After a burn-in of 25%, we used TreeAnnotator v2.1.2 [56] to generate the maximum clade credibility (MCC) tree with mean ages and 95% HPD intervals on nodes.

### Plastomic variation analysis

To assess the expansion/contraction of the IR regions, we compared the boundaries between the SC/IR and their adjacent genes by IRscope [59], and then used the results to further manually modify the annotations. To further detect the location of structural variation, we used snpEff v4.3 [60] to conduct functional annotations for the nucleotide variations in different genera distributed in different habitats.

### Positive selection analyses

We used the CODEML program in PAML v4.9 [61] to infer positive selection for 78 protein-coding genes. Pseudogenes and partial genes were excluded. Changes in the selective regime can be detected by calculating the ratio of nonsynonymous (*d*_*N*_) substitutions to synonymous (*d*_*S*_) substitutions of each plastid protein-coding gene, *ω* (*d*_*N*_/*d*_*S*_). We compared two branch models, H0: the one-ratio model (m0) that assumes to evolve under the same *ω* ratio for all branches in the phylogeny, and HA: the free-ratio model (m2) for the alternative model that assumes three independent *ω* ratio in different habitats. We used likelihood ratio tests to test each model’s fit.

### Investigating trait changes through time

We selected the 11 genes that were under positive selection (*ω* > 1) under the optimal model [62]. We coded the different amino acid sequences as characteristic states for *Ceratocephala* and *Myosurus* to infer when the genes were positively selected. To avoid the high number of species in *Ranunculus* to have an influence on the changes through time plots, a single species only was coded in *Ranunculus* as outgroup. We applied the fitDiscrete function in the R package ‘geiger’ [63] to decide which likelihood models (ER, SYM, or ARD) should be used. Simulated stochastic character maps were obtained using the *make.simmap* function in the R package ‘phytools’ under the optimal likelihood model [64]. Then we used sampled stochastic map character histories to generate ‘changes through time’ plots showing the mean number of changes and the mean rate of changes per time unit.

We further applied the ‘phytools’ function *make.era.map* to generate and fit models in which the rate of change of the composite trait varied between different times on the reference tree. Based on Westerhold et al. [23], temporal boundaries were chosen: (1) the EOT (*ca.* 34 Ma), (2) the OMB (*ca. *23.03 Ma), (3) the oMCO (*ca.* 17 Ma), (4) the mMCT (*ca.* 13.9 Ma), (5) the first major glacial event in the Northern Hemisphere (M2; *ca.* 3.3 Ma). By examining the three models (ARD, ER, SYM) and analyzing the significant differences, we selected when the rate of variation in genes changed. The likelihood ratio test (LRT) was used to compare different heterogeneous rate models for each of the genes under positive selection.

### Supplementary Information

**Supplementary Material 1.**

**Supplementary Material 2.**

## Data Availability

All sequences in this study are available in the National Center for Biotechnology Information (NCBI) (https://www.ncbi.nlm.nih.gov/nuccore/), with GenBank accession numbers (PP155434–PP155438) shown in Table S1.

## Abbreviations

ARD: All-rates-different likelihood model
BS: Bootstrap support
EOT: Eocene–Oligocene Transition
ER: Equal-rates likelihood model
GC3s: GC-content at third codon position
HPD: Highest posterior density
IRs: Inverted repeats
LRT: Likehood Ratio Test
oMCO: Onset of Miocene Climate Optimum
LSC: Long single copy
M2: The first major glacial event in the Northern Hemisphere
MCC: Maximum clade credibility
MCMC: Markov chain Monte Carlo
ML: Maximum likelihood
OMB: Oligocene–Miocene boundary
SSC: Small single copy
SYM: Symmetrical likelihood model
ca.: Circa
mMCT: Middle Miocene Climate Transition
