# Supplementary material for: Comparative plastome analysis of the sister genera Ceratocephala and Myosurus (Ranunculaceae) reveals signals of adaptive evolution to arid and aquatic environments
Source: BMC Plant Biol. 2024 Mar 20;24:202. doi: 10.1186/s12870-024-04891-2 (PMC10953084; doi:10.1186/s12870-024-04891-2)
Supplement: Supplementary file 1 — Supplementary Material 1. [file 12870_2024_4891_MOESM1_ESM.pdf]

Supplementary Information

Additional file 1:

Fig. S1. Total length of the LSC, IRs, and SSC sequences in the Ranunculeae plastomes examined in this study. IR, inverted repeats; SSC, small single-copy; LSC, large single-copy.

Fig. S2. Plastomes of seven species from four genera of Ranunculeae. (a) *Ficaria verna*, (b) *Halerpestes sarmentosa*, (c) *Oxygraphis glacialis*, (d) four species of *Ranunculus*. The genes inside and outside of the circle are transcribed in clockwise and counterclockwise directions, respectively. Genes belonging to different functional groups are shown in different colors. The thick lines indicate the extent of IR_A and IR_B that separate the genomes into SSC and LSC regions. IR, inverted repeats; SSC, small single-copy; LSC, large single-copy.

Fig. S3. Scatter plot of GC3s contents of protein-coding genes of Ranunculeae plastomes. GC3s, G or C in the third codon positions.

Fig. S4. The plastid phylogenomic tree of Ranunculeae. Maximum likelihood bootstrap support values are shown above the branches.

Fig. S5. Comparison of the LSC, IRs, and SSC region boundaries of plastomes within Ranunculeae. IR, inverted repeats; SSC, small single-copy; LSC, large single-copy.

Fig. S6. Character reconstructions for amino acid sites of the eleven genes under positive selection. Amino acid sites are listed in Table S7 in details. Character states

of amino acid sequences were coded A, B, C, and D, as referred to Table S7. Genes positively selected in *Ceratocephala* and *Myosurus* are in red; genes positively selected in *Ceratocephala* are in blue; genes positively selected in *Myosurus* are in green.

Fig. S7. Changes through time plots generated from sampled stochastic character maps for the eleven genes under positive selection. For each gene the mean rate of changes per unit time is shown. Gene names are given above the plots and their colors are the same with those in Fig. S6. The five dashed lines in each graph represented 34, 23.03, 17, 13.9 and 3.3 Ma, respectively; the colored line indicates the significant rate change point. Asterisk indicates a significant difference ($p < 0.05$; see Table S8 for details).

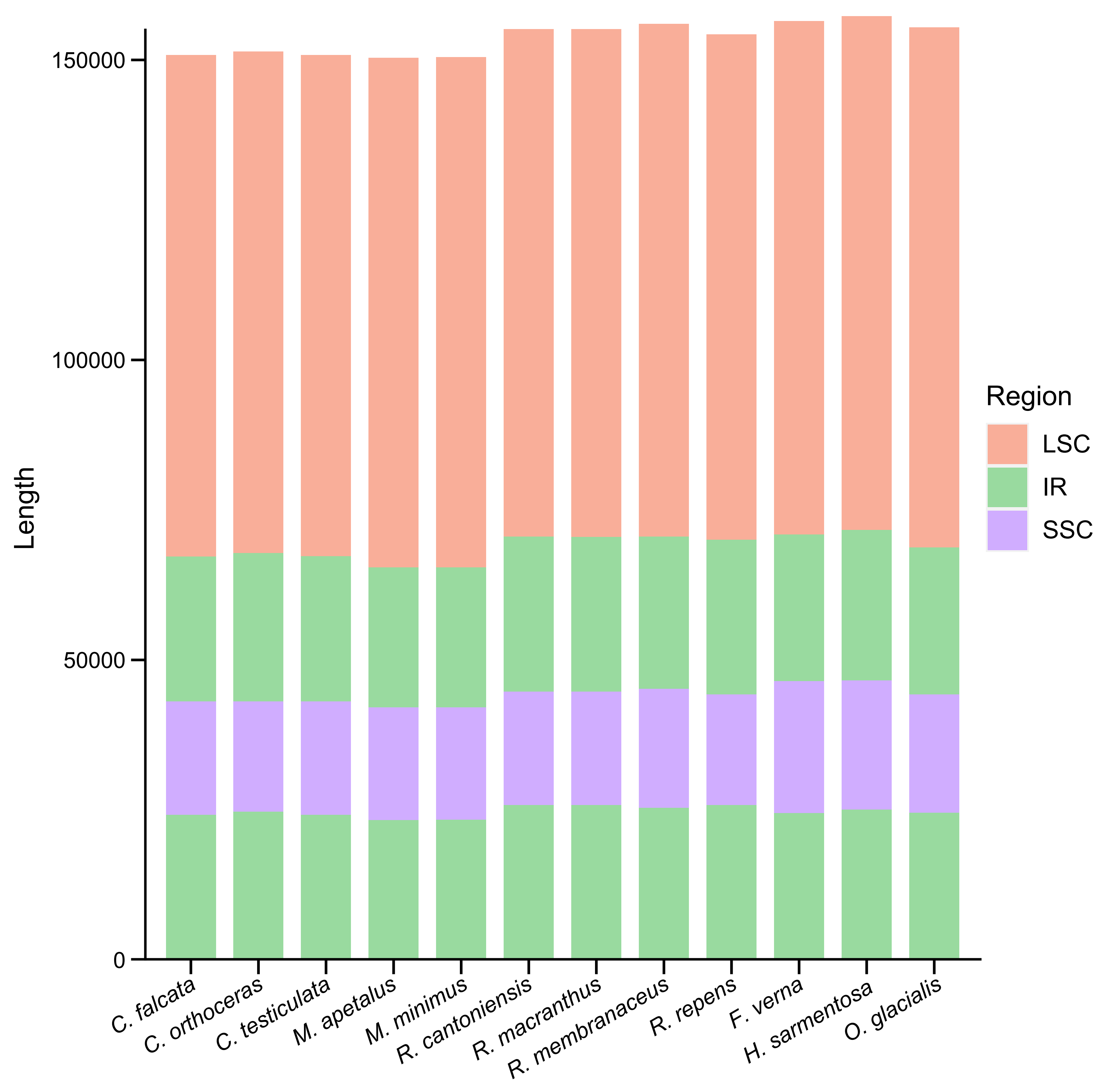

Length
150000
100000
50000
0
C. falcata
C. orthoceras
C. testiculata
M. apetalus
M. minimus
R. cantoniensis
R. macranthus
R. membranaceus
R. repens
F. verna
H. sarmentosa
O. glacialis
Region
LSC
IR
SSC

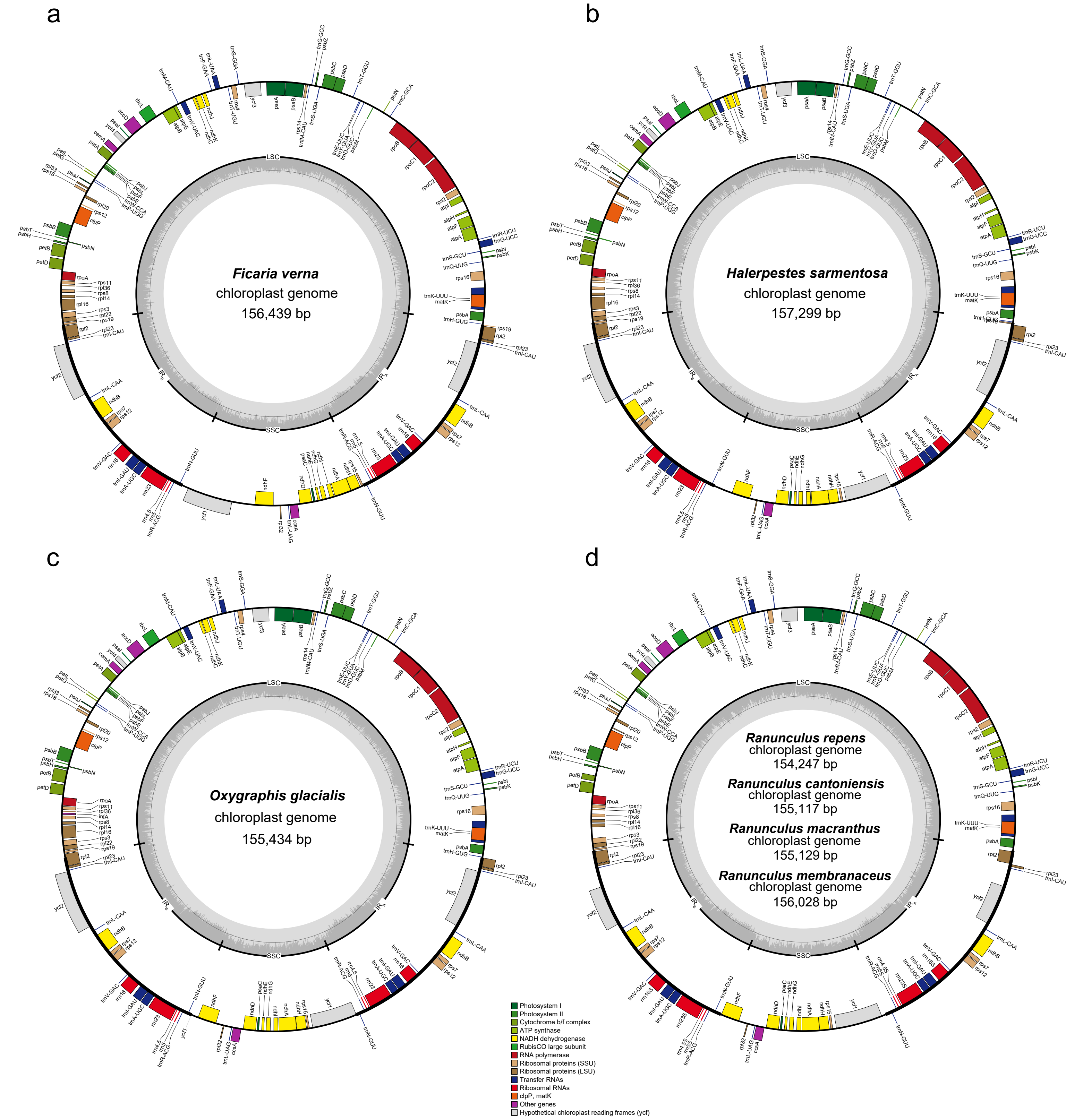
a
Ficaria verna
chloroplast genome
156,439 bp
b
Halerpestes sarmentosa
chloroplast genome
157,299 bp
c
Oxygraphis glacialis
chloroplast genome
155,434 bp
d
Ranunculus repens
chloroplast genome
154,247 bp
Ranunculus cantoniensis
chloroplast genome
155,117 bp
Ranunculus macranthus
chloroplast genome
155,129 bp
Ranunculus membranaceus
chloroplast genome
156,028 bp
LSC
SSC
Photosystem I
Photosystem II
Cytochrome b/f complex
ATP synthase
NADH dehydrogenase
RubisCO large subunit
RNA polymerase
Ribosomal proteins (SSU)
Ribosomal proteins (LSU)
Transfer RNAs
Ribosomal RNAs
clpP, matK
Other genes
Hypothetical chloroplast reading frames (ycf)

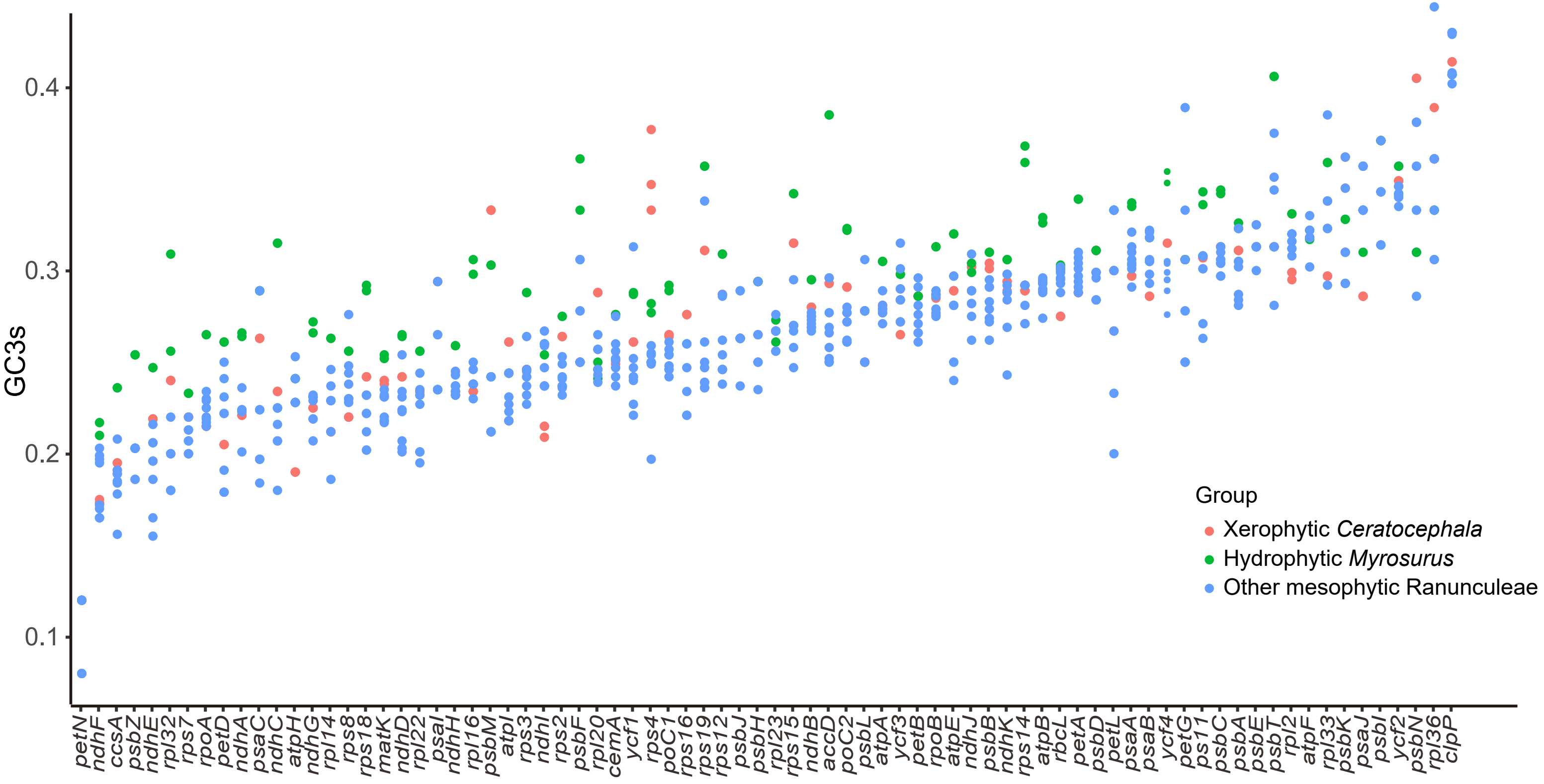
GC3s
0.1
0.2
0.3
0.4
Group
Xerophytic *Ceratocephala*
Hydrophytic *Myrosurus*
Other mesophytic Ranunculeae
petN ndhF ccsA psbZ ndhE rpl32 rps7 rpoA petD ndhA psaC ndhC atpH ndhG rpl14 rps8 rps18 matK ndhD rpl22 psaI ndhH rpl16 psbM atpI rps3 ndhI rps2 psbF rpl20 cemA ycf1 rps4 poC1 rps16 rps19 rps12 psbJ psbH rpl23 rps15 ndhB accD poC2 psbL atpA ycf3 petB rpoB atpE ndhJ psbB ndhK rps14 atpB rbcL petA psbD petL psaA psaB ycf4 petG ps11 psbC psbA psbE psbT rpl2 atpF rpl33 psbK psaJ psbI ycf2 psbN rpl36 clpP

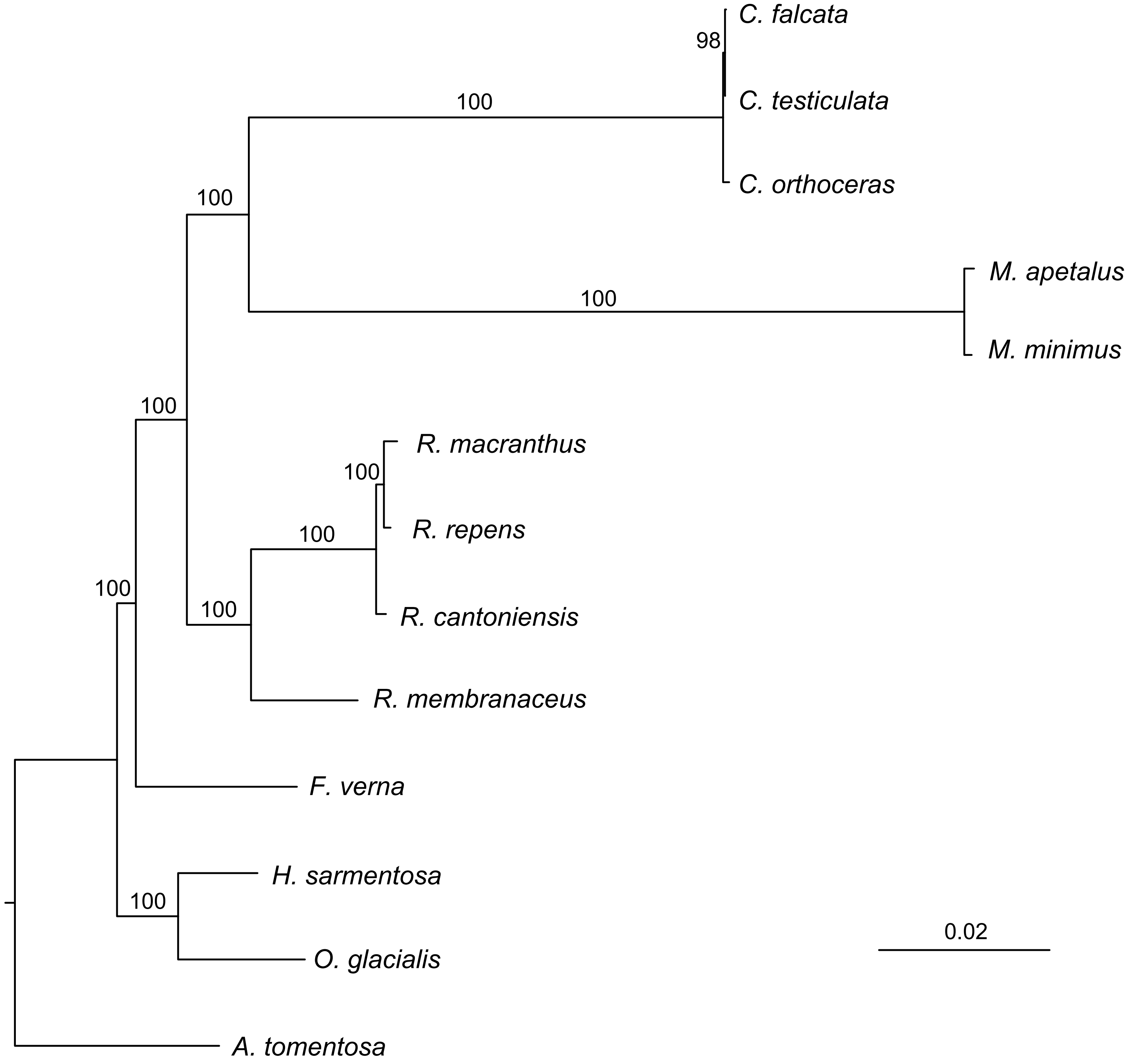
C. falcata
98
C. testiculata
100
C. orthoceras
100
M. apetalus
100
M. minimus
100
R. macranthus
100
R. repens
100
R. cantoniensis
100
100
R. membranaceus
F. verna
H. sarmentosa
100
O. glacialis
0.02
A. tomentosa

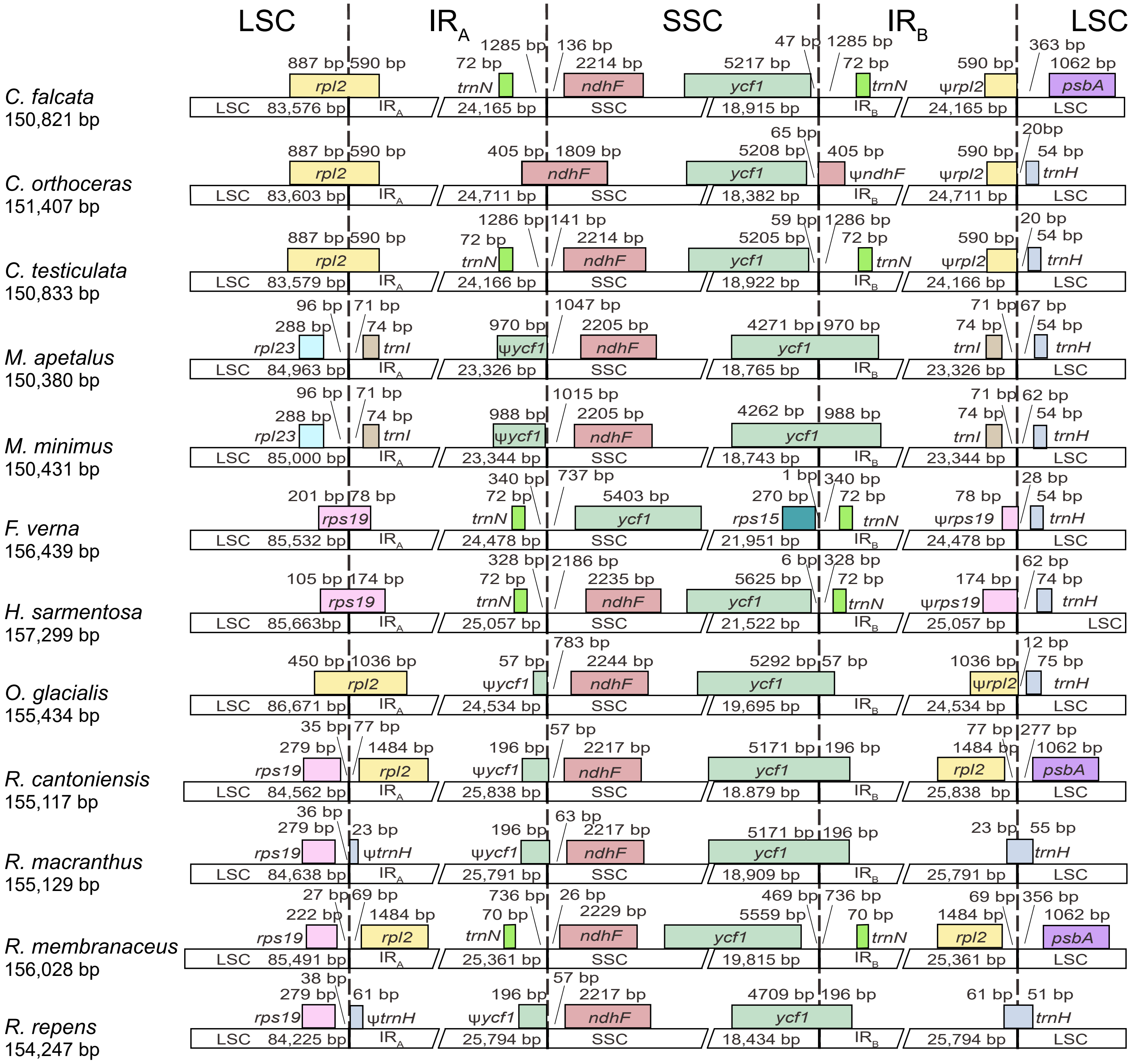

LSC
IRA
SSC
IRB
LSC
C. falcata
150,821 bp
887 bp
590 bp
rpl2
1285 bp
72 bp
trnN
136 bp
2214 bp
ndhF
5217 bp
ycf1
47 bp
1285 bp
72 bp
trnN
590 bp
ψrpl2
363 bp
1062 bp
psbA
LSC 83,576 bp
IRA
24,165 bp
SSC
18,915 bp
IRB
24,165 bp
LSC
C. orthoceras
151,407 bp
887 bp
590 bp
rpl2
405 bp
1809 bp
ndhF
65 bp
5208 bp
ycf1
405 bp
ψndhF
590 bp
ψrpl2
20bp
54 bp
trnH
LSC 83,603 bp
IRA
24,711 bp
SSC
18,382 bp
IRB
24,711 bp
LSC
C. testiculata
150,833 bp
887 bp
590 bp
rpl2
1286 bp
72 bp
trnN
141 bp
2214 bp
ndhF
59 bp
5205 bp
ycf1
1286 bp
72 bp
trnN
590 bp
ψrpl2
20 bp
54 bp
trnH
LSC 83,579 bp
IRA
24,166 bp
SSC
18,922 bp
IRB
24,166 bp
LSC
M. apetalus
150,380 bp
96 bp
288 bp
rpl23
71 bp
74 bp
trnI
970 bp
ψycf1
1047 bp
2205 bp
ndhF
4271 bp
970 bp
ycf1
71 bp
74 bp
trnI
67 bp
54 bp
trnH
LSC 84,963 bp
IRA
23,326 bp
SSC
18,765 bp
IRB
23,326 bp
LSC
M. minimus
150,431 bp
96 bp
288 bp
rpl23
71 bp
74 bp
trnI
988 bp
ψycf1
1015 bp
2205 bp
ndhF
4262 bp
988 bp
ycf1
71 bp
74 bp
trnI
62 bp
54 bp
trnH
LSC 85,000 bp
IRA
23,344 bp
SSC
18,743 bp
IRB
23,344 bp
LSC
F. verna
156,439 bp
201 bp
78 bp
rps19
340 bp
72 bp
trnN
737 bp
5403 bp
ycf1
1 bp
270 bp
rps15
340 bp
72 bp
trnN
78 bp
ψrps19
28 bp
54 bp
trnH
LSC 85,532 bp
IRA
24,478 bp
SSC
21,951 bp
IRB
24,478 bp
LSC
H. sarmentosa
157,299 bp
105 bp
174 bp
rps19
328 bp
72 bp
trnN
2186 bp
2235 bp
ndhF
6 bp
5625 bp
ycf1
328 bp
72 bp
trnN
174 bp
ψrps19
62 bp
74 bp
trnH
LSC 85,663bp
IRA
25,057 bp
SSC
21,522 bp
IRB
25,057 bp
LSC
O. glacialis
155,434 bp
450 bp
1036 bp
rpl2
57 bp
ψycf1
783 bp
2244 bp
ndhF
5292 bp
57 bp
ycf1
1036 bp
ψrpl2
12 bp
75 bp
trnH
LSC 86,671 bp
IRA
24,534 bp
SSC
19,695 bp
IRB
24,534 bp
LSC
R. cantoniensis
155,117 bp
35 bp
279 bp
rps19
77 bp
1484 bp
rpl2
196 bp
ψycf1
57 bp
2217 bp
ndhF
5171 bp
196 bp
ycf1
77 bp
1484 bp
rpl2
277 bp
1062 bp
psbA
LSC 84,562 bp
IRA
25,838 bp
SSC
18.879 bp
IRB
25,838 bp
LSC
R. macranthus
155,129 bp
36 bp
279 bp
rps19
23 bp
ψtrnH
196 bp
ψycf1
63 bp
2217 bp
ndhF
5171 bp
196 bp
ycf1
23 bp
55 bp
trnH
LSC 84,638 bp
IRA
25,791 bp
SSC
18,909 bp
IRB
25,791 bp
LSC
R. membranaceus
156,028 bp
27 bp
222 bp
rps19
69 bp
1484 bp
rpl2
736 bp
70 bp
trnN
26 bp
2229 bp
ndhF
469 bp
5559 bp
ycf1
736 bp
70 bp
trnN
69 bp
1484 bp
rpl2
356 bp
1062 bp
psbA
LSC 85,491 bp
IRA
25,361 bp
SSC
19,815 bp
IRB
25,361 bp
LSC
R. repens
154,247 bp
38 bp
279 bp
rps19
61 bp
ψtrnH
196 bp
ψycf1
57 bp
2217 bp
ndhF
4709 bp
196 bp
ycf1
61 bp
51 bp
trnH
LSC 84,225 bp
IRA
25,794 bp
SSC
18,434 bp
IRB
25,794 bp
LSC

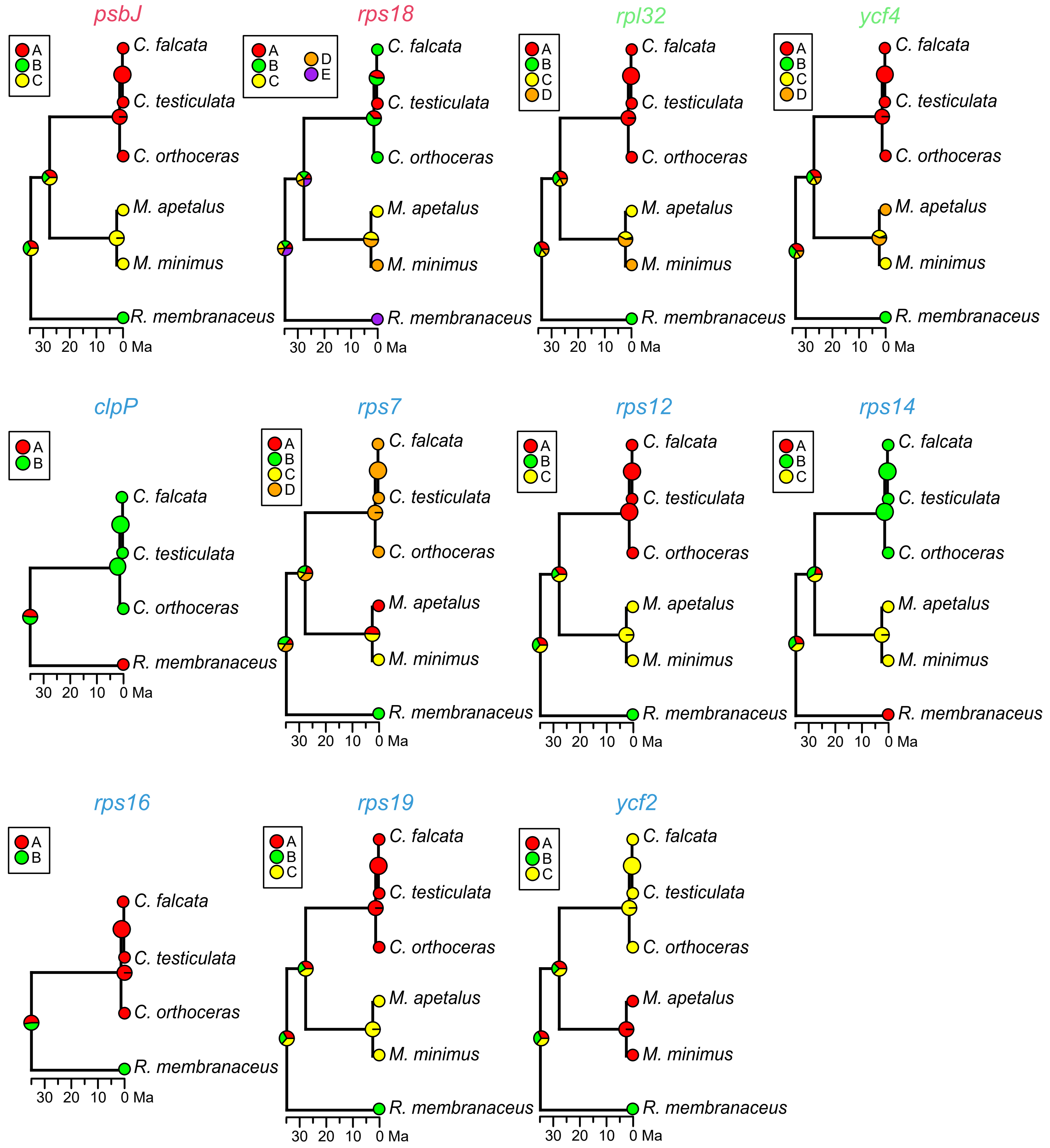

psbJ
rps18
rpl32
ycf4
clpP
rps7
rps12
rps14
rps16
rps19
ycf2
A
B
C
D
E
C. falcata
C. testiculata
C. orthoceras
M. apetalus
M. minimus
R. membranaceus
30 20 10 0 Ma

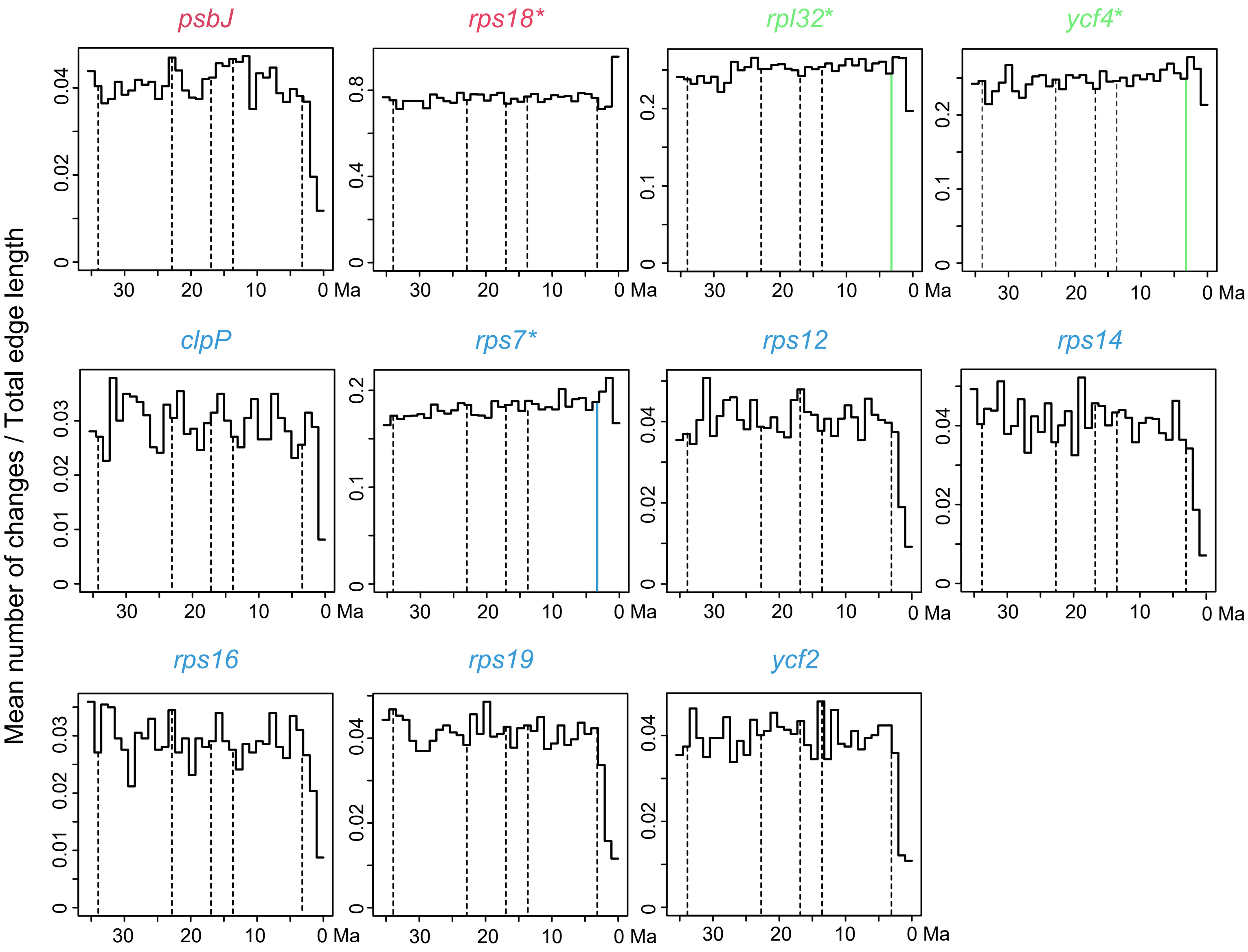

psbJ
rps18*
rpl32*
ycf4*
clpP
rps7*
rps12
rps14
rps16
rps19
ycf2
Mean number of changes / Total edge length
30
20
10
0 Ma
