# Supplementary material for: Comparative plastome analysis of the sister genera Ceratocephala and Myosurus (Ranunculaceae) reveals signals of adaptive evolution to arid and aquatic environments
Source: BMC Plant Biol. 2024 Mar 20;24:202. doi: 10.1186/s12870-024-04891-2 (PMC10953084; doi:10.1186/s12870-024-04891-2)
Supplement: Supplementary file 2 — Supplementary Material 2. [file 12870_2024_4891_MOESM2_ESM.pdf]

Additional file 2:

Table S1. Summary of characteristics of plastomic sequences of Ranunculeae used in this study.

Species	GenBank accession number	Voucher	Total genome size (GC content)	LSC size (GC content)	IR size (GC content)	SSC size (GC content)	No. total gene (unique gene)	No. protein coding gene (unique gene)	No. tRNA gene (unique gene)	No. rRNA gene (unique gene)	No. pseudogene
Ceratocephala falcata (L.) Pers.	MK253464	L Xie 2016003	150821 (38.42%)	83576 (36.76%)	24165 (43.69%)	18915 (32.32%)	130 (112)	85 (79)	37 (29)	8 (4)	1
Ceratocephalus orthoceras DC.	PP155435	PG 19930603	151407 (38.38%)	83603 (36.75%)	24711 (43.36%)	18382 (32.40%)	131 (113)	84 (79)	37 (30)	8 (4)	2
Ceratocephala testiculata (Crantz) Besser	PP155434	PG 20070625	150833 (38.42%)	83579 (36.75%)	24166 (43.69%)	18922 (32.33%)	130 (113)	84 (79)	37 (30)	8 (4)	1
Myosurus apetalus Gay	PP155437	PG 19860608	150380 (39.80%)	84963 (38.58%)	23326 (44.16%)	18765 (34.46%)	127 (111)	80 (76)	37 (30)	8 (4)	2
Myosurus minimus L.	PP155438	PG 20080502	150431 (39.79%)	85000 (38.56%)	23344 (44.17%)	18743 (34.44%)	127 (111)	80 (76)	37 (30)	8 (4)	2
Ficaria verna Huds.	PP155436	PG 20050412	156439 (38.36%)	85532 (36.65%)	24478 (43.64%)	21951 (33.25%)	130 (112)	84 (78)	37 (30)	8 (4)	1
Halerpestes sarmentosa (Adams) Kom.	MK253457	FS2015001	157299 (37.86%)	85663 (36.05%)	25057 (43.40%)	21522 (32.15%)	130 (112)	84 (78)	37 (30)	8 (4)	1
Oxygraphis glacialis (Fisch. ex DC.) Bunge	NC_041538	ZR11-016	155434 (37.75%)	86671 (35.98%)	24534 (43.31%)	19695 (31.69%)	131 (113)	83 (78)	37 (30)	8 (4)	3
Ranunculus cantoniensis DC.	NC_045920	JJU180701	155117 (37.90%)	84562 (36.01%)	25838 (43.48%)	18879 (31.10%)	129 (112)	84 (78)	35 (29)	8 (4)	2
Ranunculus macranthus Scheele	NC_008796	Rubeson et al.	155129 (37.88%)	84638 (36.00%)	25791 (43.49%)	18909 (31.01%)	132 (113)	84 (78)	38 (30)	8 (4)	3
Ranunculus membranaceus Royle	NC_065303	Ren et al.	156028 (37.91%)	85491 (36.10%)	25361 (43.48%)	19815 (31.45%)	128 (111)	84 (78)	36 (29)	8 (4)	0
Ranunculus repens L.	NC_036976	M Dann 2015/14	154247 (37.94%)	84225 (36.03%)	25794 (43.49%)	18434 (31.08%)	131 (112)	84 (78)	38 (30)	8 (4)	2
Outgroups											
Anemone tomentosa (Maxim.) Pei	NC_039451	H J Liu I-1080	160945 (37.57%)	81585 (35.63%)	31201 (41.91%)	16958 (30.95%)	134 (110)	86 (74)	36 (29)	8 (4)	4

Table S2. Functional annotations for nucleotide variants among the plastomes of *Ceratocephala*, *Myosurus*, and *Ranunculus*.

Region	Functional class	Variation type	Count
All three genera (*Ceratocephala*, *Myosurus*, and *Ranunculus*)			
Exon	Missense		2174
		Missense variant	2164
		stop_lost	10
	Nonsense		41
		stop_gained	41
	Silent		3366
		stop_retained_variant	12
		synonymous_variant	3354
Intron		intron_variant	11,650
Intergenic		intergenic_region	8419
downstream		downstream_gene_variant	52,419
upstream		upstream_gene_variant	51,569
Ceratocephala vs. Myosurus			
Exon	Missense		3198
		Missense variant	3156
		stop_lost	42
	Nonsense		91
		stop_gained	91
	Silent		2495
		stop_retained_variant	8
		synonymous_variant	2487
Intron		intron_variant	18,746
Intergenic		intergenic_region	5
downstream		downstream_gene_variant	48,494
upstream		upstream_gene_variant	48,386
Ceratocephala vs. Ranunculus			
Exon	Missense		2558
		missense_variant	2533
		stop_lost	25
	Nonsense		42
		stop_gained	42
	Silent		2291
		stop_retained_variant	10
		synonymous_variant	2281
Intron		intron_variant	19,390
Intergenic		intergenic_region	NA
downstream		downstream_gene_variant	51,428
upstream		upstream_gene_variant	51,280
Myosurus vs. Ranunculus			
Exon	Missense		3378

		missense_variant	3351
		stop_lost	27
	Nonsense		113
		stop_gained	113
	Silent		2734
		stop_retained_variant	10
		synonymous_variant	2724
Intron		intron_variant	18,360
Intergenic		intergenic_region	NA
downstream		downstream_gene_variant	55,065
upstream		upstream_gene_variant	56,844

Table S3. Summary of characteristics of genes of the plastomes in *Ceratocephala*, *Myosurus*, and *Ranunculus*.

Gene Groupe	Gene	*Ceratocephala*				*Myosurus*				*Ranunculus*			
		No. copy	GC of gene	No. introns	Size of exon	No. copy	GC of gene	No. introns	Size of exon	No. copy	GC of gene	No. introns	Size of exon
Subunit Acetyl- CoA-Carboxylate	*accD*	1	37.46%	0	1,188	1	42.85%	0	1,650	1	34.81%	0	1,491
ATP synthase	*atpA*	1	41.54%	0	1,524	1	42.45%	0	1,524	1	41.67%	0	1,524
	atpB	1	43.49%	0	1,497	1	44.42%	0	1,497	1	43.42%	0	1,497
	atpE	1	41.35%	0	399	1	42.36%	0	399	1	40.85%	0	399
	atpF	1	36.23%	1	555	1	36.59%	1	555	1	35.38%	1	552
	atpH	1	44.72%	0	246	1	45.53%	0	246	1	45.53%	0	246
	atpI	1	38.58%	0	744	1	38.31%	0	744	1	37.23%	0	744
c-type cytochrome synthesis gene	*ccsA*	1	32.61%	0	972	1	34.77%	0	972	1	31.35%	0	957
Envelop membrane protein	*cemA*	1	32.02%	0	762	1	32.32%	0	690	1	30.29%	0	690
ATP-dependent protease subunit gene	*clpP*	1	41.16%	0	741	NA	NA	NA	NA	1	36.73%	2	606
Translational initiation factor	*infA*	1	34.46%	0	177	NA	NA	NA	NA	NA	NA	NA	NA
Maturase	*matK*	1	32.68%	0	1,530	1	33.66%	0	1,536	1	30.51%	0	1,524
NADH oxidoreductase	*ndhA*	1	34.92%	1	1092	1	36.25%	1	1,092	1	33.75%	1	1,092
	ndhB	2	38.22%	1	1533	2	38.71%	1	1,533	2	37.91%	1	1,533
	ndhC	1	37.74%	0	363	1	41.05%	0	363	1	37.19%	0	363
	ndhD	1	35.86%	0	1,503	1	36.59%	0	1,503	1	34.26%	0	1,509
	ndhE	1	33.01%	0	306	1	34.31%	0	306	1	32.03%	0	306
	ndhF	1	32.34%	0	2,214	1	33.74%	0	2,205	1	30.77%	0	2,217
	ndhG	1	34.83%	0	534	1	36.14%	0	534	1	35.39%	0	534
	ndhH	1	38.58%	0	1,182	1	40.36%	0	1,182	1	38.92%	0	1,182
	ndhI	1	35.17%	0	543	1	36.85%	0	540	1	36.10%	0	543
	ndhJ	1	41.09%	0	477	1	41.72%	0	477	1	40.46%	0	477
	ndhK	1	40.06%	0	684	1	40.79%	0	684	1	39.77%	0	684

Cytochrome b6/f complex	*petA*	1	39.11%	0	969	1	40.87%	0	969	1	39.42%	0	969
	petB	1	36.13%	1	648	1	38.40%	1	648	1	40.90%	0-1	648
	petD	1	36.36%	1	510	1	38.00%	1	510	1	36.22%	1	510
	petG	1	35.96%	0	114	1	36.84%	0	114	1	35.09%	0	114
	petL	1	37.50%	0	96	1	35.42%	0	96	1	37.50%	0	96
	petN	1	40.00%	0	90	1	41.11%	0	90	1	38.89%	0	90
Photosystem I	*psaA*	1	43.19%	0	2,253	1	44.34%	0	2,253	1	43.23%	0	2,253
	psaB	1	41.68%	0	2,205	1	42.68%	0	2,205	1	42.81%	0	2,205
	psaC	1	43.09%	0	246	1	43.90%	0	246	1	41.06%	0	246
	psaI	1	36.04%	0	111	1	35.14%	0	111	1	36.04%	0	111
	psaJ	1	38.52%	0	135	1	39.26%	0	135	1	40.74%	0	135
Photosystem II	*psbA*	1	42.56%	0	1,062	1	43.03%	0	1,062	1	41.81%	0	1,062
	psbB	1	44.73%	0	1,527	1	45.45%	0	1,527	1	44.34%	0	1,527
	psbC	1	44.73%	0	1,422	1	46.06%	0	1,422	1	44.37%	0	1,422
	psbD	1	43.97%	0	1,062	1	43.79%	0	1,062	1	43.50%	0	1,062
	psbE	1	41.27%	0	252	1	41.67%	0	252	1	42.06%	0	252
	psbF	1	40.00%	0	120	1	44.17%	0	120	1	40.00%	0	120
	psbH	1	41.44%	0	222	1	40.99%	0	222	1	40.09%	0	222
	psbI	1	39.64%	0	111	1	39.64%	0	111	1	38.74%	0	111
	psbJ	1	42.28%	0	123	1	43.09%	0	123	1	42.28%	0	123
	psbK	1	36.02%	0	186	1	33.33%	0	186	1	33.87%	0	186
	psbL	1	30.77%	0	117	1	31.62%	0	117	1	31.62%	0	117
	psbM	1	34.29%	0	105	1	33.33%	0	105	1	31.43%	0	105
	psbN	1	45.45%	0	132	1	43.18%	0	132	1	43.94%	0	132
	psbT	1	36.27%	0	102	1	38.24%	0	102	1	36.27%	0	102
	psbZ	1	33.86%	0	189	1	35.45%	0	189	1	33.86%	0	189
Rubisco large subunit	*rbcL*	1	43.77%	0	1,428	1	44.68%	0	1,428	1	44.40%	0	1,428

Ribosomal proteins (LSU)	*rpl2*	1	41.44%	1	825	1	43.00%	1	825	2	42.52%	1	825
	rpl14	1	38.48%	0	369	1	39.84%	0	369	1	37.94%	0	369
	rpl16	1	36.43%	1	414	1	46.81%	0	408	1	36.18%	0-1	408
	rpl20	1	39.66%	0	348	1	37.22%	0	360	1	37.50%	0	360
	rpl22	1	35.71%	0	546	1	36.85%	0	540	1	34.81%	0	540
	rpl23	2	38.30%	0	282	1	37.50%	0	288	2	38.65%	0	282
	rpl32	1	34.57%	0	162	1	32.18%	0	174	1	31.48%	0	162
	rpl33	1	35.35%	0	198	1	37.37%	0	198	1	35.32%	0	201
	rpl36	1	42.11%	0	114	1	42.11%	0	114	1	42.11%	0	114
RNA polymerase	*rpoA*	1	34.39%	0	1,044	1	36.62%	0	1,035	1	34.87%	0	1,041
	rpoB	1	39.81%	0	3,213	1	41.07%	0	3,219	1	39.99%	0	3,213
	rpoC1	1	38.53%	1	2043	1	40.31%	0	2,034	1	37.98%	1	2,043
	rpoC2	1	38.92%	0	4,155	1	40.32%	0	4,137	1	37.06%	0	4,131
Ribosomal proteins (SSU)	*rps2*	1	40.37%	0	696	1	39.62%	0	732	1	38.54%	0	711
	rps3	1	36.07%	0	657	1	37.96%	0	648	1	36.09%	0	654
	rps4	1	35.42%	0	96	1	38.19%	0	618	1	39.11%	0	606
	rps7	2	41.88%	0	468	2	41.03%	0	468	2	39.96%	0	468
	rps8	1	37.34%	0	399	1	39.01%	0	405	1	37.59%	0	399
	rps11	1	47.52%	0	423	1	49.16%	0	417	1	47.96%	0	417
	rps12	2	40.00%	1	399	2	40.21%	1	399	2	39.72%	2	372
	rps14	1	40.92%	0	303	1	42.40%	0	375	1	40.92%	0	303
	rps15	1	31.87%	0	273	1	34.19%	0	234	1	32.23%	0	273
	rps16	1	36.77%	1	237	NA	NA	NA	NA	1	34.59%	1	237
	rps18	1	32.41%	0	108	1	36.84%	0	285	1	34.97%	0	306
	rps19	1	37.23%	0	282	1	38.52%	0	135	1	36.20%	0	279
Conserved Open reading frames	*ycf1*	1	32.22%	0	5,208	1	34.99%	0	5,250	1	36.73%	0	4,905
	ycf2	2	39.23%	0	6,375	2	39.92%	0	6,024	2	38.42%	0	6,900

	ycf3	1	38.22%	2	507	1	38.93%	2	507	1	37.42%	2	507
	ycf4	1	41.11%	0	579	1	42.46%	0	570	1	40.00%	0	555
rRNA genes	*rrn4.5*	2	49.04%	0	104	2	48.54%	0	103	2	48.54%	0	103
	rrn5	2	52.07%	0	121	2	52.07%	0	121	2	52.07%	0	121
	rrn16	2	56.20%	0	1,491	2	56.27%	0	1,491	2	56.74%	0	1,491
	rrn23	2	54.95%	0	2,810	2	54.89%	0	2,811	2	55.12%	0	2,810
tRNA genes	$trnA^{UGC}$	2	51.66%	1	73	2	51.66%	1	73	1-2	51.43%	1	73
	$trnC^{GCA}$	1	61.97%	0	71	1	63.38%	0	71	1	59.15%	0	71
	$trnD^{GUC}$	1	63.51%	0	74	1	61.64%	0	73	1	63.51%	0	73
	$trnE^{UUC}$	1	58.90%	0	73	1	57.53%	0	73	1	58.90%	0	73
	$trnF^{GAA}$	1	50.68%	0	73	1	52.05%	0	73	1	50.68%	0	73
	$trnfM^{CAU}$	1	54.79%	0	73	1	52.70%	0	74	1	56.76%	0	74
	$trnG^{GCC}$	1	52.11%	0	71	1	52.11%	0	71	1	52.11%	0	71
	$trnG^{UCC}$	1	34.61%	1	71	1	36.27%	1	71	1	33.72%	1	71
	$trnH^{GUG}$	1	59.26%	0	54	1	57.41%	0	54	1-2	56.86%	0-1	54
	$trnI^{CAU}$	2	45.95%	0	74	2	44.59%	0	74	2	45.95%	0	74
	$trnI^{GAU}$	2	49.32%	1	72	2	49.75%	1	72	2	49.95%	1	72
	$trnK^{UUU}$	1	32.95%	1	72	1	34.26%	1	72	1	31.50%	1	72
	$trnL^{CAA}$	2	49.38%	0	81	2	49.38%	0	81	2	49.38%	0	81
	$trnL^{UAA}$	1	37.02%	1	85	1	36.68%	1	85	1	36.11%	1	85
	$trnL^{UAG}$	1	56.25%	0	80	1	56.25%	0	80	1	56.25%	0	80
	$trnM^{CAU}$	1	39.73%	0	73	1	39.73%	0	73	1	41.10%	0	73
	$trnN^{GUU}$	2	52.78%	0	72	2	54.17%	0	72	2	52.78%	0	72
	$trnP^{UGG}$	1	47.30%	0	74	1	48.65%	0	74	1	47.30%	0	74
	$trnQ^{UUG}$	1	58.33%	0	72	1	56.16%	0	73	1	62.50%	0	73
	$trnR^{ACG}$	2	62.16%	0	74	2	62.16%	0	74	2	62.16%	0	74
	$trnR^{UCU}$	1	43.06%	0	72	1	43.06%	0	72	1	43.06%	0	72

trnS^{GCU}	1	50.00%	0	88	1	48.86%	0	88	1	51.14%	0	88
trnS^{GGA}	1	52.87%	0	87	1	51.72%	0	87	1	52.87%	0	87
trnS^{UGA}	1	47.31%	0	93	1	47.73%	0	88	1	47.31%	0	88
trnT^{GGU}	1	50.00%	0	72	1	50.00%	0	72	1	50.00%	0	72
trnT^{UGU}	1	53.42%	0	73	1	50.68%	0	73	1	53.42%	0	73
trnV^{GAC}	2	48.61%	0	72	2	50.00%	0	72	2	51.39%	0	72
trnV^{UAC}	1	39.60%	1	73	1	39.84%	1	73	1	38.90%	1	73
trnW^{CCA}	1	51.35%	0	74	1	50.00%	0	74	1	51.35%	0	74
trnY^{GUA}	1	53.57%	0	84	1	53.57%	0	84	1	53.57%	0	84

Table S4. The d_N, d_S and ω values calculated under the one-ratio model (m0).

Gene	*C. falcata*	*C. orthoceras*	*C. testiculata*	*M. apetalus*	*M. minimus*	*F. verna*	*H. sarmentosa*	*O. glacialis*	*R. cantoniensis*	*R. macranthus*	*R. membranaceus*	*R. repens*	*A. tomentosa*
d_N													
accD	0.0007	0.0007	0	0	0	0.0123	0.0055	0.0078	0	0.0008	0.0064	0	0.0135
atpA	0	0.0002	0	0.0006	0	0.0026	0.0015	0.0018	0.0001	0.0002	0.0035	0.0002	0.0020
atpB	0	0.0005	0	0.0006	0.0002	0.0029	0.0019	0.0022	0	0.0002	0.0011	0.0003	0.0048
atpE	0	0	0	0	0	0.0029	0.0012	0.0011	0.0006	0.0006	0.0041	0.0006	0.0078
atpF	0	0	0	0.0010	0	0.0094	0.0010	0.0041	0	0	0.0073	0	0.0116
atpH	0	0	0	0	0	0.0005	0.0000	0.0010	0	0	0.0003	0	0.0008
atpI	0	0	0	0	0	0.0022	0.0009	0.0010	0	0	0.0013	0	0.0028
ccsA	0	0.0007	0	0.0029	0.0006	0.0224	0.0158	0.0233	0.0035	0.0062	0.0187	0.0007	0.0251
clpP	0	0	0	NA	NA	0.0154	0.0030	0.0092	0	0.0030	0.0033	0.0002	NA
cemA	0	0	0	0.0020	0	0.0200	0.0040	0.0100	0	0.0010	0.0018	0.0010	0.0195
matK	0	0.0005	0	0.0030	0	0.0259	0.0103	0.0186	0.0020	0.0020	0.0194	0.0005	0.0381
ndhA	0	0	0	0.0011	0	0.0080	0.0063	0.0088	0.0007	0.0004	0.0078	0.0011	NA
ndhB	0	0	0	0	0	0	0.0004	0.0004	0	0.0008	0.0004	0.0004	0.0035
ndhC	0	0	0	0	0	0.0038	0.0009	0.0009	0	0	0.0029	0.0018	0.0067
ndhD	0	0	0	0.0010	0.0005	0.0101	0.0064	0.0091	0.0006	0.0005	0.0080	0.0007	0.0125
ndhE	0	0.0012	0	0	0.0012	0.0118	0.0077	0.0092	0.0012	0	0.0068	0	0.0058
ndhF	0	0.0006	0	0.0010	0.0006	0.0196	0.0149	0.0173	0.0021	0.0017	0.0137	0	0.0290
ndhG	0.0011	0	0	0	0.0031	0.0111	0.0087	0.0133	0	0	0.0151	0	0.0172
ndhH	0	0.0004	0	0.0007	0.0002	0.0067	0.0031	0.0020	0.0004	0.0004	0.0032	0	0.0086
ndhI	0.0007	0	0	0	0.0007	0.0120	0.0009	0.0063	0	0.0021	0.0043	0	0.0075
ndhJ	0	0	0	0.0006	0	0.0067	0.0037	0.0019	0	0.0006	0.0027	0.0006	0.0049
ndhK	0	0.0006	0	0	0	0.0079	0.0032	0.0103	0	0.0006	0.0026	0	0.0121
petA	0	0	0	0.0003	0	0.0068	0.0018	0.0052	0	0.0010	0.0042	0	0.0094
petB	0	0	0	0	0	0.0014	0.0006	0.0009	0	0	0.0009	0	0.0018
petD	0	0	0	0	0	0.0064	0.0010	0.0028	0.0005	0.0005	0.0074	0	0.0079

petG	0	0	0	0	0	0	0	0.0009	0	0	0.0019	0	0.0034
petL	0.0044	0	0	0	0	0.0148	0.0048	0	0	0	0.0048	0.0047	0.0123
petN	0	0	0	0	0	0	0	0	0	0	0	0	0
psaA	0	0	0	0.0001	0.0001	0.0007	0.0004	0.0006	0	0	0.0008	0	0.0021
psaB	0	0	0	0	0.0001	0.0006	0.0005	0.0004	0	0.0001	0.0004	0	0.0008
psaC	0	0	0	0	0	0	0	0	0	0	0	0	0
psaI	0	0	0	0	0	0	0	0.0050	0	0	0.0104	0	0.0074
psaJ	0	0	0	0	0.0017	0.0071	0	0.0017	0	0	0	0.0017	0.0094
psbA	0	0	0	0	0.0001	0.0006	0.0006	0.0004	0.0001	0.0001	0.0005	0	0.0002
psbB	0	0.0001	0	0	0	0.0013	0.0009	0.0013	0	0.0002	0.0013	0	0.0035
psbC	0	0	0	0.0001	0	0.0007	0.0008	0.0010	0	0.0001	0.0005	0.0001	0.0014
psbD	0	0	0	0	0.0001	0.0006	0.0005	0.0003	0	0	0.0002	0	0.0009
psbE	0	0	0	0	0	0	0	0.0003	0.0003	0	0	0	0.0017
psbF	0	0	0	0.0009	0	0	0	0.0009	0	0	0.0009	0	0.0012
psbH	0	0	0	0	0.0017	0.0035	0.0018	0.0036	0	0	0.0059	0	0.0140
psbI	0	0	0	0	0	0	0	0.0000	0	0	0	0	0
psbJ	0	0	0	0	0	0.0093	0	0.0095	0	0	0.0047	0	0
psbK	0	0.0029	0	0	0	0.0058	0.0029	0.0029	0.0029	0.0030	0.0029	0	0.0092
psbL	0	0	0	0	0	0	0	0	0	0	0	0	0
psbM	0	0	0	0	0	0	0.0012	0	0	0	0	0	0.0018
psbN	0	0	0	0	0	0.0052	0	0.0019	0	0	0.0010	0.0019	0
psbT	0	0	0	0	0	0	0	0	0	0	0	0	0
psbZ	0	0	0	0	0	0.0026	0.0053	0	0.0026	0	0	0	0.0052
rbcL	0	0.0002	0	0.0001	0	0.0016	0.0005	0.0016	0.0002	0.0002	0.0019	0.0003	0.0043
rpl14	0	0	0	0	0	0.0043	0.0009	0.0034	0	0	0.0018	0	0.0057
rpl16	0	0	0	0	0.0011	0.0146	0.0011	0.0033	0.0011	0.0011	0.0090	0	0.0102
rpl20	0	0	0	0	0	0.0198	0.0080	0.0019	0	0	0.0059	0	0.0336
rpl22	0	0	0	0	0	0.0064	0.0074	0.0073	0.0009	0	0.0035	0	0.0069
rpl23	0	0	0	0.0022	0.0019	0.0021	0.0021	0	0	0	0	0	0.0003

rpl2	0	0.0006	0	0	0	0.0029	0	0.0029	0	0	0.0006	0	0.0066
rpl32	0	0	0	0.0137	0.0040	0.0240	0.0240	0.0091	0	0	0.0083	0	NA
rpl33	0	0	0	0	0	0.0129	0.0054	0.0052	0	0	0.0055	0	0.0123
rpl36	0	0.0004	0	0	0	0.0012	0	0.0004	0	0	0.0007	0	0.0019
rpoA	0	0.0006	0	0	0.0006	0.0163	0.0073	0.0126	0.0019	0.0013	0.0085	0	0.0001
rpoB	0	0.0002	0	0.0004	0.0001	0.0067	0.0023	0.0049	0	0.0006	0.0040	0	0.0088
rpoC1	0	0.0002	0	0.0002	0.0005	0.0056	0.0036	0.0035	0.0005	0	0.0035	0	0.0163
rpoC2	0	0.0004	0	0.0003	0	0.0111	0.0054	0.0078	0.0011	0.0006	0.0066	0.0001	0.0127
rps11	0.0014	0	0	0.0028	0	0.0080	0.0043	0.0138	0	0	0.0036	0	0.0182
rps12	0	0	0	0	0	0.0024	0.0024	0.0024	0	0	0.0219	0	0.0069
rps14	0	0	0	0.0021	0	0.0034	0.0022	0.0130	0	0	0.0000	0	0.0067
rps15	0	0.0028	0	0.0088	0	0.0200	0.0032	0.0082	0.0029	0	0.0192	0	0.0131
rps18	0	0	0.0086	0.0075	0.0103	0.0086	0.0174	0	0	0.0087	0	0	0.0008
rps19	0	0	0	0	0	0.0023	0.0029	0	0	0	0	0	0.0026
rps2	0	0	0	0	0.0009	0.0035	0.0056	0.0025	0	0.0018	0.0054	0	0.0184
rps3	0	0	0	0	0	0.0107	0.0106	0.0068	0.0010	0.0010	0.0073	0.0027	0.0130
rps4	NA	NA	NA	0.0020	0	0.0038	0.0043	0.0028	0	0	0.0010	0	0.0136
rps7	0	0	0	0.0018	0	0.0055	0	0.0018	0.0018	0	0	0.0018	0.0013
rps8	0	0	0	0.0011	0	0.0068	0.0034	0.0057	0	0	0.0034	0	0.0041
rps16	0	0	0	NA	NA	0.0073	0.0072	0	0	0	0.0107	0	NA
ycf1	0	0.0012	0	0	0.0018	0.0332	0.0184	0.0440	0.0012	0.0024	0.0197	0.0030	0.0299
ycf2	0	0	0	0	0	0.0064	0.0017	0.0026	0	0	0.0041	0.0006	0.0131
ycf3	0	0.0003	0	0.0003	0	0.0024	0.0010	0.0010	0	0	0.0006	0	0.0032
ycf4	0	0	0	0	0.0012	0.0087	0.0042	0.0070	0	0	0.0071	0	0.0140
d_S													
accD	0.0025	0.0025	0	0	0	0.0414	0.0187	0.0265	0.0000	0.0026	0.0217	0	0.0457
atpA	0	0.0022	0	0.0067	0	0.0297	0.0175	0.0210	0.0016	0.0022	0.0403	0.0022	0.0237
atpB	0	0.0069	0	0.0090	0.0024	0.0416	0.0277	0.0321	0.0000	0.0023	0.0162	0.0046	0.0680
atpE	0	0	0	0	0	0.0470	0.0190	0.0178	0.0091	0.0090	0.0659	0.0091	0.1245

atpF	0	0	0	0.0049	0	0.0459	0.0051	0.0201	0.0000	0	0.0357	0	0.0564
atpH	0	0	0	0	0	0.0500	0	0.1058	0.0000	0	0.0335	0	0.0892
atpI	0	0	0	0	0	0.0546	0.0222	0.0262	0.0000	0	0.0320	0	0.0692
ccsA	0	0.0028	0	0.0118	0.0025	0.0926	0.0653	0.0963	0.0143	0.0255	0.0774	0.0028	0.1036
clpP	0	0	0	NA	NA	0.0222	0.0044	0.0132	0.0000	0.0043	0.0047	0.0003	NA
cemA	0	0	0	0.0063	0	0.0648	0.0128	0.0326	0.0000	0.0032	0.0060	0.0032	0.0632
matK	0	0.0015	0	0.0088	0	0.0764	0.0303	0.0548	0.0058	0.0058	0.0573	0.0014	0.1121
ndhA	0.0002	0.0001	0	0.0080	0	0.0576	0.0454	0.0639	0.0053	0.0027	0.0561	0.0080	NA
ndhB	0	0	0	0	0	0.0000	0.0017	0.0017	0.0000	0.0033	0.0017	0.0017	0.0154
ndhC	0	0	0	0	0	0.0398	0.0090	0.0094	0.0000	0	0.0308	0.0185	0.0708
ndhD	0	0	0	0.0090	0.0043	0.0941	0.0602	0.0852	0.0052	0.0048	0.0750	0.0066	0.1169
ndhE	0	0.0118	0	0	0.0115	0.1124	0.0734	0.0869	0.0118	0	0.0643	0	0.0553
ndhF	0	0.0028	0	0.0052	0.0031	0.0987	0.0750	0.0871	0.0104	0.0083	0.0689	0	0.1459
ndhG	0.0047	0.0000	0	0	0.0139	0.0494	0.0386	0.0591	0.0000	0	0.0671	0	0.0762
ndhH	0	0.0064	0	0.0098	0.0027	0.0996	0.0455	0.0301	0.0063	0.0063	0.0476	0	0.1276
ndhI	0.0068	0	0	0	0.0066	0.1147	0.0083	0.0603	0.0000	0.0202	0.0415	0	0.0713
ndhJ	0	0	0	0.0079	0	0.0873	0.0485	0.0250	0.0000	0.0079	0.0355	0.0079	0.0641
ndhK	0	0.0044	0	0	0	0.0540	0.0220	0.0704	0.0000	0.0044	0.0178	0	0.0822
petA	0	0	0	0.0035	0	0.0720	0.0193	0.0544	0.0000	0.0106	0.0438	0	0.0992
petB	0	0	0	0	0	0.0554	0.0249	0.0376	0.0000	0	0.0385	0	0.0736
petD	0	0	0	0	0	0.0976	0.0152	0.0421	0.0072	0.0072	0.1131	0	0.1199
petG	0	0	0	0	0	0	0	0.0246	0	0	0.0507	0	0.0924
petL	0.0283	0	0	0	0	0.0954	0.0308	0	0	0	0.0307	0.0305	0.0791
petN	0	0	0	0	0	0	0	0	0	0	0.0000	0	0.0000
psaA	0	0	0	0.0031	0.0036	0.0365	0.0209	0.0301	0	0	0.0398	0.0017	0.1054
psaB	0	0	0	0	0.0036	0.0327	0.0264	0.0203	0	0.0036	0.0233	0.0018	0.0418
psaC	0	0	0	0	0	0.0400	0.0578	0.1209	0.0409	0.0411	0.0625	0	0.1454
psaI	0	0	0	0	0	0	0	0.0215	0	0	0.0448	0	0.0319
psaJ	0	0	0	0	0.0206	0.0860	0	0.0205	0	0	0	0.0205	0.1143

psbA	0	0	0	0	0.0037	0.0308	0.0306	0.0197	0.0038	0.0038	0.0271	0	0.0120
psbB	0	0.0024	0	0	0	0.0335	0.0228	0.0346	0	0.0048	0.0337	0	0.0893
psbC	0	0	0	0.0025	0	0.0226	0.0235	0.0290	0	0.0025	0.0155	0.0026	0.0411
psbD	0	0	0	0	0.0034	0.0279	0.0235	0.0112	0	0	0.0071	0	0.0410
psbE	0	0	0	0	0	0	0	0.0150	0.0151	0	0	0	0.0931
psbF	0	0	0	0.0313	0	0	0	0.0314	0	0	0.0314	0	0.0414
psbH	0	0	0	0	0.0138	0.0281	0.0147	0.0291	0	0	0.0470	0	0.1115
psbI	0	0	0	0	0	0	0.0275	0.0277	0	0	0.0295	0	0.0692
psbJ	0	0	0	0	0	0.0399	0	0.0406	0	0	0.0199	0	0.0000
psbK	0	0.0139	0	0	0	0.0272	0.0137	0.0135	0.0136	0.0139	0.0136	0	0.0436
psbL	0	0	0	0	0	0.0993	0	0.0482	0	0	0.0487	0	0.0302
psbM	0	0	0	0	0	0	0.0391	0	0	0	0.0000	0	0.0582
psbN	0	0	0	0	0	0.1445	0	0.0534	0	0	0.0267	0.0532	0.0003
psbT	0	0	0	0	0	0	0.0388	0.0387	0	0	0	0	0.0506
psbZ	0	0	0	0	0	0.0140	0.0283	0	0.0141	0	0	0	0.0276
rbcL	0	0.0051	0	0.0025	0	0.0341	0.0103	0.0340	0.0036	0.0048	0.0398	0.0054	0.0894
rpl14	0	0	0	0	0	0.0500	0.0100	0.0398	0	0	0.0207	0	0.0659
rpl16	0	0	0	0	0.0072	0.0966	0.0071	0.0219	0.0072	0.0073	0.0594	0	0.0677
rpl20	0	0	0	0	0	0.0654	0.0264	0.0062	0	0	0.0195	0	0.1111
rpl22	0	0	0	0	0	0.0483	0.0555	0.0549	0.0066	0	0.0266	0	0.0520
rpl23	0	0	0	0.0111	0.0099	0.0105	0.0105	0	0	0	0.0000	0	0.0015
rpl2	0	0.0039	0	0	0	0.0195	0	0.0195	0	0	0.0039	0	0.0442
rpl32	0.0001	0	0	0.0626	0.0182	0.1102	0.1099	0.0419	0.0002	0	0.0383	0.0001	NA
rpl33	0	0	0	0	0	0.0746	0.0313	0.0299	0	0	0.0319	0	0.0716
rpl36	0	0.0331	0	0	0	0.1089	0	0.0347	0	0	0.0676	0	0.1756
rpoA	0	0.0027	0	0	0.0027	0.0697	0.0311	0.0540	0.0082	0.0054	0.0364	0	0.0002
rpoB	0	0.0020	0	0.0031	0.0008	0.0524	0.0183	0.0382	0	0.0049	0.0317	0	0.0686
rpoC1	0	0.0014	0	0.0015	0.0028	0.0334	0.0218	0.0207	0.0028	0	0.0208	0	0.0972
rpoC2	0	0.0018	0	0.0012	0	0.0438	0.0215	0.0307	0.0042	0.0024	0.0262	0.0006	0.0500

rps11	0.0054	0	0	0.0107	0	0.0301	0.0161	0.0520	0	0	0.0134	0	0.0686
rps12	0	0	0	0	0	0.0041	0.0041	0.0041	0	0	0.0376	0	0.0118
rps14	0	0	0	0.0078	0	0.0128	0.0081	0.0485	0	0	0.0000	0	0.0250
rps15	0	0.0100	0	0.0310	0	0.0704	0.0112	0.0290	0.0101	0	0.0676	0	0.0461
rps18	0	0	0.0143	0.0124	0.0171	0.0144	0.0288	0	0	0.0144	0	0	0.0013
rps19	0	0	0	0	0	0.0970	0.1219	0	0	0	0	0	0.1116
rps2	0	0	0	0	0.0035	0.0137	0.0219	0.0100	0	0.0071	0.0213	0	0.0726
rps3	0	0	0	0	0	0.0482	0.0474	0.0308	0.0044	0.0044	0.0327	0.0122	0.0585
rps4	NA	NA	NA	0.0094	0	0.0180	0.0206	0.0131	0.0002	0	0.0049	0.0001	0.0648
rps7	0	0	0	0.0036	0	0.0109	0	0.0036	0.0036	0	0	0.0036	0.0025
rps8	0	0	0	0.0074	0	0.0450	0.0223	0.0376	0	0	0.0228	0	0.0271
rps16	0.0002	0.0001	0	NA	NA	0.0148	0.0147	0	0.0005	0.0003	0.0217	0.0004	NA
ycf1	0	0.0026	0	0	0.0039	0.0709	0.0393	0.0940	0.0026	0.0052	0.0421	0.0064	0.0639
ycf2	0	0	0	0	0	0.0085	0.0022	0.0034	0	0	0.0055	0.0008	0.0173
ycf3	0	0.0080	0	0.0080	0	0.0604	0.0239	0.0248	0	0	0.0160	0	0.0804
ycf4	0	0	0	0	0.0042	0.0311	0.0152	0.0252	0	0	0.0256	0	0.0502
ω													
accD	0.2962	0.2962	0.2962	0.2962	0.2962	0.2962	0.2962	0.2962	0.2962	0.2962	0.2962	0.2962	0.2962
atpA	0.0861	0.0861	0.0861	0.0861	0.0861	0.0861	0.0861	0.0861	0.0861	0.0861	0.0861	0.0861	0.0861
atpB	0.0700	0.0700	0.0700	0.0700	0.0700	0.0700	0.0700	0.0700	0.0700	0.0700	0.0700	0.0700	0.0700
atpE	0.0625	0.0625	0.0625	0.0625	0.0625	0.0625	0.0625	0.0625	0.0625	0.0625	0.0625	0.0625	0.0625
atpF	0.2054	0.2054	0.2054	0.2054	0.2054	0.2054	0.2054	0.2054	0.2054	0.2054	0.2054	0.2054	0.2054
atpH	0.0092	0.0092	0.0092	0.0092	0.0092	0.0092	0.0092	0.0092	0.0092	0.0092	0.0092	0.0092	0.0092
atpI	0.0399	0.0399	0.0399	0.0399	0.0399	0.0399	0.0399	0.0399	0.0399	0.0399	0.0399	0.0399	0.0399
ccsA	0.2421	0.2421	0.2421	0.2421	0.2421	0.2421	0.2421	0.2421	0.2421	0.2421	0.2421	0.2421	0.2421
clpP	0.6943	0.6943	0.6943	NA	NA	0.6943	0.6943	0.6943	0.6943	0.6943	0.6943	0.6943	NA
matK	0.3395	0.3395	0.3395	0.3395	0.3395	0.3395	0.3395	0.3395	0.3395	0.3395	0.3395	0.3395	0.3395
ndhA	0.1384	0.1384	0.1384	0.1384	0.1384	0.1384	0.1384	0.1384	0.1384	0.1384	0.1384	0.1384	NA
ndhB	0.2279	0.2279	0.2279	0.2279	0.2279	0.2279	0.2279	0.2279	0.2279	0.2279	0.2279	0.2279	0.2279

ndhC	0.0949	0.0949	0.0949	0.0949	0.0949	0.0949	0.0949	0.0949	0.0949	0.0949	0.0949	0.0949	0.0949
ndhD	0.1071	0.1071	0.1071	0.1071	0.1071	0.1071	0.1071	0.1071	0.1071	0.1071	0.1071	0.1071	0.1071
ndhE	0.1053	0.1053	0.1053	0.1053	0.1053	0.1053	0.1053	0.1053	0.1053	0.1053	0.1053	0.1053	0.1053
ndhF	0.1987	0.1987	0.1987	0.1987	0.1987	0.1987	0.1987	0.1987	0.1987	0.1987	0.1987	0.1987	0.1987
ndhG	0.2253	0.2253	0.2253	0.2253	0.2253	0.2253	0.2253	0.2253	0.2253	0.2253	0.2253	0.2253	0.2253
ndhH	0.0672	0.0672	0.0672	0.0672	0.0672	0.0672	0.0672	0.0672	0.0672	0.0672	0.0672	0.0672	0.0672
ndhI	0.1048	0.1048	0.1048	0.1048	0.1048	0.1048	0.1048	0.1048	0.1048	0.1048	0.1048	0.1048	0.1048
ndhJ	0.0763	0.0763	0.0763	0.0763	0.0763	0.0763	0.0763	0.0763	0.0763	0.0763	0.0763	0.0763	0.0763
ndhK	0.1466	0.1466	0.1466	0.1466	0.1466	0.1466	0.1466	0.1466	0.1466	0.1466	0.1466	0.1466	0.1466
petA	0.0950	0.0950	0.0950	0.0950	0.0950	0.0950	0.0950	0.0950	0.0950	0.0950	0.0950	0.0950	0.0950
petB	0.0244	0.0244	0.0244	0.0244	0.0244	0.0244	0.0244	0.0244	0.0244	0.0244	0.0244	0.0244	0.0244
petD	0.0656	0.0656	0.0656	0.0656	0.0656	0.0656	0.0656	0.0656	0.0656	0.0656	0.0656	0.0656	0.0656
petG	0.0371	0.0371	0.0371	0.0371	0.0371	0.0371	0.0371	0.0371	0.0371	0.0371	0.0371	0.0371	0.0371
petL	0.1551	0.1551	0.1551	0.1551	0.1551	0.1551	0.1551	0.1551	0.1551	0.1551	0.1551	0.1551	0.1551
petN	0.0001	0.0001	0.0001	0.0001	0.0001	0.0001	0.0001	0.0001	0.0001	0.0001	0.0001	0.0001	0.0001
psaA	0.0200	0.0200	0.0200	0.0200	0.0200	0.0200	0.0200	0.0200	0.0200	0.0200	0.0200	0.0200	0.0200
psaB	0.0182	0.0182	0.0182	0.0182	0.0182	0.0182	0.0182	0.0182	0.0182	0.0182	0.0182	0.0182	0.0182
psaC	0.0001	0.0001	0.0001	0.0001	0.0001	0.0001	0.0001	0.0001	0.0001	0.0001	0.0001	0.0001	0.0001
psaI	0.2321	0.2321	0.2321	0.2321	0.2321	0.2321	0.2321	0.2321	0.2321	0.2321	0.2321	0.2321	0.2321
psaJ	0.0821	0.0821	0.0821	0.0821	0.0821	0.0821	0.0821	0.0821	0.0821	0.0821	0.0821	0.0821	0.0821
psbA	0.0198	0.0198	0.0198	0.0198	0.0198	0.0198	0.0198	0.0198	0.0198	0.0198	0.0198	0.0198	0.0198
psbB	0.0389	0.0389	0.0389	0.0389	0.0389	0.0389	0.0389	0.0389	0.0389	0.0389	0.0389	0.0389	0.0389
psbC	0.0331	0.0331	0.0331	0.0331	0.0331	0.0331	0.0331	0.0331	0.0331	0.0331	0.0331	0.0331	0.0331
psbD	0.0229	0.0229	0.0229	0.0229	0.0229	0.0229	0.0229	0.0229	0.0229	0.0229	0.0229	0.0229	0.0229
psbE	0.0180	0.0180	0.0180	0.0180	0.0180	0.0180	0.0180	0.0180	0.0180	0.0180	0.0180	0.0180	0.0180
psbF	0.0301	0.0301	0.0301	0.0301	0.0301	0.0301	0.0301	0.0301	0.0301	0.0301	0.0301	0.0301	0.0301
psbH	0.1253	0.1253	0.1253	0.1253	0.1253	0.1253	0.1253	0.1253	0.1253	0.1253	0.1253	0.1253	0.1253
psbI	0.0001	0.0001	0.0001	0.0001	0.0001	0.0001	0.0001	0.0001	0.0001	0.0001	0.0001	0.0001	0.0001
psbJ	0.2333	0.2333	0.2333	0.2333	0.2333	0.2333	0.2333	0.2333	0.2333	0.2333	0.2333	0.2333	0.2333

psbK	0.2118	0.2118	0.2118	0.2118	0.2118	0.2118	0.2118	0.2118	0.2118	0.2118	0.2118	0.2118	0.2118
psbL	0.0001	0.0001	0.0001	0.0001	0.0001	0.0001	0.0001	0.0001	0.0001	0.0001	0.0001	0.0001	0.0001
psbM	0.0301	0.0301	0.0301	0.0301	0.0301	0.0301	0.0301	0.0301	0.0301	0.0301	0.0301	0.0301	0.0301
psbN	0.0362	0.0362	0.0362	0.0362	0.0362	0.0362	0.0362	0.0362	0.0362	0.0362	0.0362	0.0362	0.0362
psbT	0.0001	0.0001	0.0001	0.0001	0.0001	0.0001	0.0001	0.0001	0.0001	0.0001	0.0001	0.0001	0.0001
psbZ	0.1875	0.1875	0.1875	0.1875	0.1875	0.1875	0.1875	0.1875	0.1875	0.1875	0.1875	0.1875	0.1875
rbcL	0.0479	0.0479	0.0479	0.0479	0.0479	0.0479	0.0479	0.0479	0.0479	0.0479	0.0479	0.0479	0.0479
rpl14	0.0865	0.0865	0.0865	0.0865	0.0865	0.0865	0.0865	0.0865	0.0865	0.0865	0.0865	0.0865	0.0865
rpl16	0.1508	0.1508	0.1508	0.1508	0.1508	0.1508	0.1508	0.1508	0.1508	0.1508	0.1508	0.1508	0.1508
rpl20	0.3025	0.3025	0.3025	0.3025	0.3025	0.3025	0.3025	0.3025	0.3025	0.3025	0.3025	0.3025	0.3025
rpl22	0.1331	0.1331	0.1331	0.1331	0.1331	0.1331	0.1331	0.1331	0.1331	0.1331	0.1331	0.1331	0.1331
rpl23	0.1956	0.1956	0.1956	0.1956	0.1956	0.1956	0.1956	0.1956	0.1956	0.1956	0.1956	0.1956	0.1956
rpl2	0.1487	0.1487	0.1487	0.1487	0.1487	0.1487	0.1487	0.1487	0.1487	0.1487	0.1487	0.1487	0.1487
rpl32	0.2182	0.2182	0.2182	0.2182	0.2182	0.2182	0.2182	0.2182	0.2182	0.2182	0.2182	0.2182	NA
rpl33	0.1725	0.1725	0.1725	0.1725	0.1725	0.1725	0.1725	0.1725	0.1725	0.1725	0.1725	0.1725	0.1725
rpl36	0.0106	0.0106	0.0106	0.0106	0.0106	0.0106	0.0106	0.0106	0.0106	0.0106	0.0106	0.0106	0.0106
rpoA	0.2344	0.2344	0.2344	0.2344	0.2344	0.2344	0.2344	0.2344	0.2344	0.2344	0.2344	0.2344	0.2344
rpoB	0.1276	0.1276	0.1276	0.1276	0.1276	0.1276	0.1276	0.1276	0.1276	0.1276	0.1276	0.1276	0.1276
rpoC1	0.1672	0.1672	0.1672	0.1672	0.1672	0.1672	0.1672	0.1672	0.1672	0.1672	0.1672	0.1672	0.1672
rpoC2	0.2535	0.2535	0.2535	0.2535	0.2535	0.2535	0.2535	0.2535	0.2535	0.2535	0.2535	0.2535	0.2535
rps11	0.2656	0.2656	0.2656	0.2656	0.2656	0.2656	0.2656	0.2656	0.2656	0.2656	0.2656	0.2656	0.2656
rps12	0.5826	0.5826	0.5826	0.5826	0.5826	0.5826	0.5826	0.5826	0.5826	0.5826	0.5826	0.5826	0.5826
rps14	0.2672	0.2672	0.2672	0.2672	0.2672	0.2672	0.2672	0.2672	0.2672	0.2672	0.2672	0.2672	0.2672
rps15	0.2838	0.2838	0.2838	0.2838	0.2838	0.2838	0.2838	0.2838	0.2838	0.2838	0.2838	0.2838	0.2838
rps18	0.6016	0.6016	0.6016	0.6016	0.6016	0.6016	0.6016	0.6016	0.6016	0.6016	0.6016	0.6016	0.6016
rps19	0.0237	0.0237	0.0237	0.0237	0.0237	0.0237	0.0237	0.0237	0.0237	0.0237	0.0237	0.0237	0.0237
rps2	0.2536	0.2536	0.2536	0.2536	0.2536	0.2536	0.2536	0.2536	0.2536	0.2536	0.2536	0.2536	0.2536
rps3	0.2226	0.2226	0.2226	0.2226	0.2226	0.2226	0.2226	0.2226	0.2226	0.2226	0.2226	0.2226	0.2226
rps4	NA	NA	NA	0.2099	0.2099	0.2099	0.2099	0.2099	0.2099	0.2099	0.2099	0.2099	0.2099

rps7	0.5012	0.5012	0.5012	0.5012	0.5012	0.5012	0.5012	0.5012	0.5012	0.5012	0.5012	0.5012	0.5012
rps8	0.1511	0.1511	0.1511	0.1511	0.1511	0.1511	0.1511	0.1511	0.1511	0.1511	0.1511	0.1511	0.1511
rps16	0.4929	0.4929	0.4929	NA	NA	0.4929	0.4929	0.4929	0.4929	0.4929	0.4929	0.4929	NA
ycf1	0.4682	0.4682	0.4682	0.4682	0.4682	0.4682	0.4682	0.4682	0.4682	0.4682	0.4682	0.4682	0.4682
ycf2	0.7560	0.7560	0.7560	0.7560	0.7560	0.7560	0.7560	0.7560	0.7560	0.7560	0.7560	0.7560	0.7560
ycf3	0.0402	0.0402	0.0402	0.0402	0.0402	0.0402	0.0402	0.0402	0.0402	0.0402	0.0402	0.0402	0.0402
ycf4	0.2783	0.2783	0.2783	0.2783	0.2783	0.2783	0.2783	0.2783	0.2783	0.2783	0.2783	0.2783	0.2783

Table S5. The d_N, d_S and ω values calculated under the free-ratio model (m2).

Gene	*C. falcata*	*C. orthoceras*	*C. testiculata*	*M. apetalus*	*M. minimus*	*F. verna*	*H. sarmentosa*	*O. glacialis*	*R. cantoniensis*	*R. macranthus*	*R. membranaceus*	*R. repens*	*A. tomentosa*
d_N													
accD	0.001064	0.001065	0.000001	0.000001	0.000001	0.009479	0.004287	0.006097	0.000001	0.000596	0.004963	0.000001	0.018124
atpA	0	0.000185	0	0.000917	0.000001	0.002013	0.001187	0.001426	0.00011	0.000152	0.002748	0.000151	0.007482
atpB	0	0.000695	0	0.000692	0.000177	0.002412	0.001606	0.001864	0	0.000133	0.00094	0.000268	0.001421
atpE	0	0	0	0	0	0.002411	0.000972	0.000912	0.000466	0.000462	0.003381	0.000465	0.004810
atpF	0.000001	0.000001	0.000001	0.001661	0.000001	0.007783	0.000855	0.003404	0.000001	0.000001	0.006019	0.000001	0.015332
atpH	0	0	0	0	0	0.000005	0	0.000011	0	0	0.000003	0	0.000006
atpI	0	0	0	0	0	0.002632	0.001076	0.001263	0	0	0.001545	0	0.001543
ccsA	0.000001	0.000616	0.000001	0.002432	0.000489	0.023433	0.016553	0.024271	0.003625	0.006449	0.019699	0.000716	0.031356
clpP	0.000001	0.000001	0.000001	NA	NA	0.008215	0.001593	0.004887	0.000001	0.001538	0.001662	0.000106	NA
cemA	0.000001	0.000001	0.000001	0.002284	0.000001	0.018554	0.003655	0.009298	0.000001	0.000906	0.001698	0.000906	0.024350
matK	0.000001	0.000544	0.000001	0.003054	0.000001	0.025312	0.010043	0.018181	0.001931	0.001916	0.018971	0.000479	0.030087
ndhA	0	0	0	0.001461	0.000001	0.008158	0.006467	0.00908	0.000756	0.000379	0.008017	0.001143	NA
ndhB	0.000001	0.000001	0.000001	0	0	0.000001	0.000377	0.000377	0.000001	0.000755	0.000375	0.000378	0.000001
ndhC	0	0	0	0	0	0.003722	0.000842	0.000876	0	0	0.002869	0.001731	0.005709
ndhD	0	0	0	0.001326	0.000659	0.009486	0.006027	0.008532	0.000517	0.000482	0.007483	0.000662	0.012461
ndhE	0.000001	0.002560	0.000001	0	0.001260	0.009937	0.006475	0.007691	0.001039	0	0.005676	0	0.000731
ndhF	0.000001	0.000602	0.000001	0.000986	0.000585	0.019505	0.014814	0.01717	0.002051	0.001648	0.013601	0.000001	0.025303
ndhG	0.001215	0.000001	0.000001	0.000001	0.002737	0.011241	0.008774	0.013414	0.000001	0.000001	0.015262	0.000001	0.021826
ndhH	0	0.000507	0	0.000538	0.000149	0.006708	0.003057	0.002029	0.000426	0.000424	0.0032	0	0.011032
ndhI	0.000946	0.000001	0.000001	0	0.000569	0.012132	0.000889	0.006378	0	0.002136	0.004411	0	0.004979
ndhJ	0	0	0	0.001330	0.000001	0.004066	0.002246	0.001154	0	0.000363	0.001625	0.000362	0.005464
ndhK	0.000001	0.000671	0.000001	0	0	0.008984	0.003675	0.011708	0.000001	0.000735	0.002969	0.000001	0.000308
petA	0	0	0	0.000300	0	0.006905	0.001858	0.005212	0	0.001015	0.00419	0	0.007673

petB	0	0	0	0	0	0.001320	0.000594	0.000896	0	0	0.000917	0	0.002451
petD	0.000001	0.000001	0.000001	0	0	0.005853	0.000920	0.002557	0.000434	0.000434	0.006779	0	0.004947
petG	0.000001	0.000001	0.000001	0	0	0	0	0.000003	0	0	0.000005	0	0.000008
petL	0.002885	0	0	0	0	0.018057	0.005875	0.000001	0.000001	0.000001	0.005848	0.005828	0.008366
petN	0	0	0	0	0	0	0	0	0	0	0	0	0
psaA	0	0	0	0.000057	0.000066	0.000775	0.000443	0.000639	0	0	0.000844	0.000036	0.000533
psaB	0	0	0	0	0.000148	0.000380	0.000307	0.000237	0	0.000042	0.000271	0.000021	0.000870
psaC	0	0	0	0	0	0.000004	0.000006	0.000012	0.000004	0.000004	0.000006	0	0.000006
psaI	0.000001	0.000001	0.000001	0	0	0.000001	0.000001	0.004575	0.000001	0.000001	0.009543	0.000001	0.004103
psaJ	0.000001	0.000001	0.000001	0	0.000002	0.005514	0	0.001308	0	0	0	0.001308	0.004426
psbA	0	0	0	0	0.000071	0.000699	0.000696	0.000448	0.000087	0.000086	0.000617	0	0.001243
psbB	0	0.000098	0	0	0	0.001376	0.000938	0.001423	0	0.000197	0.001385	0	0.003593
psbC	0	0	0	0.000176	0	0.000586	0.000614	0.000757	0	0.000066	0.000403	0.000067	0.001440
psbD	0	0	0	0	0.000145	0.000470	0.000395	0.000189	0	0	0.000119	0	0.001813
psbE	0.000001	0.000001	0.000001	0	0	0	0	0.000002	0.000002	0	0	0	0.000010
psbF	0	0	0	0.002900	0	0	0	0.000003	0	0	0.000003	0	0.000010
psbH	0	0	0	0	0.001487	0.003727	0.001952	0.003849	0.000001	0.000001	0.006224	0.000001	0.014627
psbI	0	0	0	0	0	0	0.000003	0.000003	0	0	0.000003	0	0.000004
psbJ	0.000002	0.000002	0.000002	0.000002	0.000002	0.007715	0.000001	0.007856	0.000001	0.000001	0.00386	0.000001	0.007904
psbK	0.000001	0.003843	0.000001	0.000001	0.000001	0.005264	0.002660	0.002616	0.002635	0.002696	0.002619	0.000001	0.006240
psbL	0	0	0	0	0	0.000010	0	0.000005	0	0	0.000005	0	0.000003
psbM	0	0	0	0	0	0	0.001942	0	0	0	0	0	0.001833
psbN	0	0	0	0	0	0.004193	0	0.001545	0	0	0.000772	0.00154	0.000603
psbT	0	0	0	0	0	0	0.000004	0.000004	0	0	0	0	0.000006
psbZ	0	0	0	0	0	0.003624	0.007307	0.000001	0.003642	0.000001	0.000001	0.000001	0.000070
rbcL	0	0.000213	0	0	0	0.002101	0.000637	0.002095	0.000221	0.000298	0.002452	0.000332	0.003962
rpl14	0	0	0	0.000001	0.000001	0.001252	0.000250	0.000992	0	0	0.0005250	0	0.000358

rpl16	0	0	0	0.000001	0.001295	0.014242	0.001054	0.003222	0.001059	0.001068	0.008751	0.000001	0.004884
rpl20	0.000001	0.000001	0.000001	0.000001	0.000001	0.018325	0.007409	0.001737	0.000001	0.000001	0.005477	0.000001	0.023008
rpl22	0.000001	0.000001	0.000001	0	0	0.005415	0.006280	0.006224	0.000750	0	0.0029910	0	0.005549
rpl23	0	0	0	0.002127	0.001894	0.002250	0.002245	0.000001	0.000001	0.000001	0.000001	0.000001	0.002239
rpl2	0.000001	0.000549	0.000001	0.000001	0.000001	0.002650	0.000001	0.002649	0.000001	0.000001	0.000525	0.000001	0.004716
rpl32	0	0	0	0.029858	0.006062	0.013234	0.011769	0.005594	0	0	0.005476	0	NA
rpl33	0	0	0	0.000001	0.000001	0.014147	0.005929	0.005672	0.000001	0.000001	0.006026	0.000001	0.011917
rpl36	0	0.000003	0	0	0	0.001672	0	0.000534	0	0	0.00104	0	0.003348
rpoA	0.000001	0.000643	0.000001	0.000001	0.000696	0.015778	0.007043	0.012224	0.001843	0.001231	0.00823	0.000001	0.012875
rpoB	0.000001	0.000306	0.000001	0.000510	0.000142	0.005555	0.001931	0.004043	0	0.000517	0.00335	0	0.005321
rpoC1	0.000001	0.000317	0.000001	0.000282	0.000548	0.004696	0.003065	0.002918	0.000401	0.000001	0.002927	0.000001	0.009010
rpoC2	0.000001	0.000430	0.000001	0.000346	0.000001	0.010595	0.005193	0.007434	0.001008	0.000573	0.00634	0.000143	0.022065
rps11	0.002098	0.000001	0.000001	0.004199	0.000001	0.004102	0.002212	0.007116	0	0	0.001807	0	0.008692
rps12	0.000002	0.000002	0.000002	0.000001	0.000001	0.002041	0.002044	0.002049	0.000001	0.000001	0.018772	0.000001	0.001403
rps14	0.000001	0.000001	0.000001	0.002298	0.000001	0.002590	0.001625	0.009787	0.000001	0.000001	0.000001	0.000001	0.000761
rps15	0.000001	0.002581	0.000001	0.009340	0.000001	0.019906	0.003123	0.008209	0.002859	0.000001	0.019119	0.000001	0.018024
rps18	0.000001	0.000001	0.010431	0.008873	0.014126	0.000005	0.000010	0	0	0.000005	0	0	0.000005
rps19	0.000002	0.000002	0.000002	0.000001	0.000001	0.000019	0.000014	0	0	0	0	0	0.000008
rps2	0.000001	0.000001	0.000001	0.000001	0.000992	0.003211	0.005180	0.002363	0.000001	0.001671	0.005043	0.000001	0.012458
rps3	0.000001	0.000001	0.000001	0.000001	0.000001	0.007471	0.007446	0.004843	0.000683	0.000683	0.005061	0.001889	0.007635
rps4	NA	NA	NA	0.003283	0.000001	0.002483	0.002765	0.001646	0	0	0.000626	0	0.014874
rps7	0.000002	0.000002	0.000002	0.002155	0.000001	0.002423	0	0.000805	0.000802	0	0	0.000803	0.005703
rps8	0	0	0	0.001384	0.000001	0.006968	0.003462	0.005834	0.000001	0.000001	0.003527	0.000001	0.006008
rps16	0.000001	0.000001	0.000001	NA	NA	0.004600	0.004587	0.000001	0.000001	0.000001	0.006544	0.000001	NA
ycf1	0.000001	0.001253	0.000001	0.000001	0.002115	0.031069	0.017189	0.041158	0.001115	0.002251	0.018433	0.002812	0.026149
ycf2	0.000001	0.000001	0.000001	0.000001	0.000001	0.005572	0.001469	0.002229	0.000001	0.000001	0.003586	0.000555	0.005602
ycf3	0	0.000318	0	0.000527	0	0.001895	0.000750	0.000778	0	0	0.000501	0	0.002809

ycf4	0.000001	0.000001	0.000001	0.000001	0.001943	0.004393	0.002142	0.003554	0	0	0.003582	0	0.007725
d_S													
accD	0.001280	0.001281	0.000002	0.000003	0.000003	0.052933	0.023942	0.034046	0.000004	0.003326	0.027714	0.000004	0.101207
atpA	0.000004	0.002234	0.000004	0.005590	0.000004	0.031436	0.018534	0.022258	0.001722	0.002369	0.042905	0.002364	0.116814
atpB	0.000004	0.006239	0.000004	0.008872	0.002269	0.043173	0.028758	0.033375	0.000004	0.002385	0.016826	0.004792	0.025429
atpE	0.000005	0.000005	0.000005	0.000004	0.000004	0.048866	0.019712	0.018480	0.009438	0.009366	0.068533	0.009418	0.097509
atpF	0.000004	0.000004	0.000004	0.002788	0.000002	0.051780	0.005685	0.022645	0.000004	0.000004	0.040039	0.000004	0.102001
atpH	0.000005	0.000005	0.000005	0.000004	0.000004	0.051462	0.000005	0.109185	0.000005	0.000005	0.034428	0.000005	0.055885
atpI	0.000005	0.000005	0.000005	0.000005	0.000005	0.052943	0.021634	0.025405	0.000005	0.000005	0.031083	0.000005	0.031039
ccsA	0.000004	0.003010	0.000004	0.013609	0.002738	0.088409	0.062450	0.091571	0.013676	0.024332	0.074322	0.002700	0.118300
clpP	0.000001	0.000001	0.000001	NA	NA	0.046194	0.008959	0.027483	0.000003	0.008648	0.009344	0.000595	NA
cemA	0.000003	0.000003	0.000003	0.005274	0.000002	0.070263	0.013842	0.035208	0.000003	0.003432	0.006431	0.003431	0.092210
matK	0.000002	0.001246	0.000002	0.008420	0.000003	0.079171	0.031412	0.056866	0.006041	0.005993	0.059339	0.001499	0.094106
ndhA	0.000004	0.000004	0.000004	0.007004	0.000003	0.056625	0.044888	0.063020	0.005246	0.002629	0.055647	0.007936	NA
ndhB	0.000002	0.000002	0.000002	0.000004	0.000004	0.000003	0.001661	0.001661	0.000003	0.003326	0.001653	0.001663	0.000003
ndhC	0.000004	0.000004	0.000004	0.000004	0.000004	0.039938	0.009036	0.009396	0.000004	0.000004	0.030781	0.018573	0.061256
ndhD	0.000004	0.000004	0.000004	0.007659	0.003808	0.097377	0.061869	0.087587	0.005306	0.004947	0.076811	0.006795	0.127920
ndhE	0.000003	0.006785	0.000003	0.000004	0.011306	0.121025	0.078859	0.093674	0.012658	0.000005	0.069125	0.000005	0.008902
ndhF	0.000003	0.002568	0.000003	0.005424	0.003219	0.099419	0.075507	0.087516	0.010454	0.008399	0.069325	0.000004	0.128967
ndhG	0.004257	0.000003	0.000003	0.000003	0.015019	0.049184	0.038392	0.058695	0.000003	0.000003	0.066779	0.000003	0.095498
ndhH	0.000004	0.006091	0.000004	0.010231	0.002838	0.099613	0.045400	0.030136	0.006320	0.006295	0.047515	0.000005	0.163825
ndhI	0.005870	0.000004	0.000004	0.000005	0.007125	0.114016	0.008356	0.059936	0.000004	0.020078	0.041457	0.000004	0.046791
ndhJ	0.000004	0.000004	0.000004	0.005173	0.000003	0.098433	0.054361	0.027930	0.000005	0.008795	0.039345	0.008768	0.132281
ndhK	0.000004	0.004255	0.000004	0.000005	0.000005	0.050168	0.020523	0.065380	0.000004	0.004105	0.016578	0.000004	0.001723
petA	0.000004	0.000004	0.000004	0.003576	0.000004	0.071781	0.019312	0.054179	0.000004	0.010551	0.043557	0.000004	0.079758
petB	0.000005	0.000005	0.000005	0.000005	0.000005	0.055527	0.024976	0.037690	0.000005	0.000005	0.038596	0.000005	0.103109
petD	0.000003	0.000003	0.000003	0.000005	0.000005	0.098124	0.015424	0.042867	0.007277	0.007273	0.113643	0.000004	0.082931

petG	0.000003	0.000003	0.000003	0.000004	0.000004	0.000004	0.000004	0.026272	0.000004	0.000004	0.054511	0.000004	0.078676
petL	0.032183	0.000004	0.000004	0.000004	0.000004	0.084185	0.027390	0.000003	0.000003	0.000003	0.027263	0.027172	0.039003
petN	0.000005	0.000005	0.000005	0.000005	0.000005	0.000005	0.000005	0.000005	0.000005	0.000005	0.000005	0.000005	0.000005
psaA	0.000005	0.000005	0.000005	0.003146	0.003640	0.036371	0.020801	0.029993	0.000005	0.000005	0.039600	0.001696	0.025018
psaB	0.000005	0.000005	0.000005	0.000005	0.003358	0.033356	0.026921	0.020781	0.000005	0.003716	0.023786	0.001851	0.076285
psaC	0.000006	0.000006	0.000006	0.000006	0.000006	0.039999	0.057770	0.120939	0.040949	0.041116	0.062505	0.000006	0.059100
psaI	0.000002	0.000002	0.000002	0.000005	0.000005	0.000003	0.000003	0.022689	0.000003	0.000003	0.047330	0.000003	0.020350
psaJ	0.000003	0.000003	0.000003	0.000004	0.024427	0.089704	0.000004	0.021275	0.000004	0.000004	0.000004	0.021286	0.072009
psbA	0.000005	0.000005	0.000005	0.000005	0.003745	0.030482	0.030320	0.019530	0.003773	0.003767	0.026879	0.000005	0.054195
psbB	0.000004	0.002343	0.000004	0.000005	0.000005	0.033227	0.022639	0.034365	0.000004	0.004746	0.033428	0.000004	0.086747
psbC	0.000005	0.000005	0.000005	0.002240	0.000004	0.022931	0.024005	0.029601	0.000005	0.002597	0.015786	0.002602	0.056355
psbD	0.000004	0.000004	0.000004	0.000004	0.003178	0.028441	0.023906	0.011410	0.000005	0.000005	0.007227	0.000005	0.109696
psbE	0.000003	0.000003	0.000003	0.000005	0.000005	0.000005	0.000005	0.015796	0.015868	0.000005	0.000005	0.000005	0.097128
psbF	0.000005	0.000005	0.000005	0.025651	0.000004	0.000005	0.000005	0.034193	0.000005	0.000005	0.034140	0.000005	0.098785
psbH	0.000004	0.000004	0.000004	0.000004	0.014576	0.027424	0.014363	0.028324	0.000004	0.000004	0.045801	0.000004	0.107638
psbI	0.000004	0.000004	0.000004	0.000004	0.000004	0.000004	0.027487	0.027683	0.000004	0.000004	0.029538	0.000004	0.038194
psbJ	0	0	0	0	0	0.044512	0.000003	0.045324	0.000003	0.000003	0.022270	0.000003	0.045599
psbK	0.000003	0.011274	0.000003	0.000003	0.000003	0.028767	0.014536	0.014296	0.014399	0.014735	0.014312	0.000003	0.034104
psbL	0.000006	0.000006	0.000006	0.000006	0.000006	0.09931	0.000006	0.048153	0.000006	0.000006	0.048715	0.000006	0.034514
psbM	0.000005	0.000005	0.000005	0.000005	0.000005	0.000004	0.036591	0.000004	0.000004	0.000004	0.000004	0.000004	0.034527
psbN	0.000005	0.000005	0.000005	0.000004	0.000004	0.147882	0.000004	0.054479	0.000004	0.000004	0.027228	0.054320	0.021258
psbT	0.000005	0.000005	0.000005	0.000005	0.000005	0.000005	0.038801	0.038740	0.000005	0.000005	0.000005	0.000005	0.059641
psbZ	0.000005	0.000005	0.000005	0.000005	0.000005	0.011002	0.022181	0.000003	0.011057	0.000003	0.000003	0.000003	0.000213
rbcL	0.000005	0.005217	0.000005	0.002909	0.000005	0.032576	0.009871	0.032480	0.003427	0.004623	0.038021	0.005146	0.061430
rpl14	0.000005	0.000005	0.000005	0.000003	0.000003	0.061684	0.012319	0.048910	0.000006	0.000006	0.025861	0.000006	0.017649
rpl16	0.000004	0.000004	0.000004	0.000003	0.006468	0.097955	0.007248	0.022160	0.007283	0.007342	0.060185	0.000004	0.033594
rpl20	0.000002	0.000002	0.000002	0.000003	0.000003	0.070932	0.028677	0.006724	0.000003	0.000003	0.021200	0.000003	0.089059

rpl22	0.000003	0.000003	0.000003	0.000005	0.000005	0.052581	0.060979	0.060435	0.007282	0.000005	0.029046	0.000005	0.053885
rpl23	0.000006	0.000006	0.000006	0.011343	0.010103	0.009685	0.009662	0.000003	0.000003	0.000003	0.000003	0.000003	0.009636
rpl2	0.000004	0.004012	0.000004	0.000004	0.000004	0.020350	0.000004	0.020344	0.000004	0.000004	0.004034	0.000004	0.036215
rpl32	0.000005	0.000005	0.000005	0.010612	0.002155	0.165134	0.146854	0.069795	0.000005	0.000005	0.068327	0.000005	NA
rpl33	0.000005	0.000005	0.000005	0.000002	0.000002	0.069710	0.029216	0.027950	0.000004	0.000004	0.029694	0.000004	0.058720
rpl36	0.000005	0.034093	0.000005	0.000005	0.000005	0.107132	0.000005	0.034214	0.000005	0.000005	0.066649	0.000005	0.214499
rpoA	0.000003	0.002677	0.000003	0.000003	0.002491	0.072342	0.032292	0.056046	0.008452	0.005645	0.037734	0.000004	0.059031
rpoB	0.000004	0.001761	0.000004	0.002642	0.000735	0.056699	0.019710	0.041260	0.000004	0.005279	0.034191	0.000004	0.054304
rpoC1	0.000003	0.001167	0.000003	0.001331	0.002588	0.036631	0.023909	0.022766	0.003126	0.000004	0.022837	0.000004	0.070288
rpoC2	0.000003	0.001841	0.000003	0.001032	0.000003	0.045588	0.022345	0.031988	0.004339	0.002464	0.027278	0.000614	0.094940
rps11	0.003525	0.000002	0.000002	0.007033	0.000002	0.041906	0.022597	0.072705	0.000004	0.000004	0.018462	0.000004	0.088799
rps12	0	0	0	0.000001	0.000001	0.005399	0.005407	0.005420	0.000003	0.000003	0.049652	0.000003	0.003710
rps14	0.000001	0.000001	0.000001	0.007129	0.000003	0.016114	0.010108	0.060888	0.000004	0.000004	0.000004	0.000004	0.004734
rps15	0.000003	0.010870	0.000003	0.029299	0.000003	0.070963	0.011135	0.029263	0.010192	0.000003	0.068158	0.000003	0.064255
rps18	0.000001	0.000001	0.006885	0.004429	0.007052	0.049254	0.096588	0.000006	0.000006	0.048669	0.000006	0.000006	0.047046
rps19	0.000001	0.000001	0.000001	0.000003	0.000003	0.191986	0.138862	0.000007	0.000007	0.000007	0.000007	0.000007	0.077336
rps2	0.000003	0.000003	0.000003	0.000003	0.003192	0.014459	0.023325	0.010642	0.000003	0.007525	0.022708	0.000003	0.056094
rps3	0.000004	0.000004	0.000004	0.000002	0.000002	0.061364	0.061153	0.039776	0.005610	0.005610	0.041567	0.015513	0.062703
rps4	NA	NA	NA	0.004922	0.000002	0.024604	0.027397	0.016305	0.000004	0.000004	0.006199	0.000004	0.147360
rps7	0.000001	0.000001	0.000001	0.002324	0.000001	0.023103	0.000005	0.007679	0.007646	0.000005	0.000005	0.007658	0.054370
rps8	0.000005	0.000005	0.000005	0.006549	0.000003	0.044380	0.022052	0.037155	0.000004	0.000004	0.022466	0.000004	0.038264
rps16	0.000001	0.000001	0.000001	NA	NA	0.025108	0.025033	0.000004	0.000004	0.000004	0.035717	0.000004	NA
ycf1	0.000002	0.002465	0.000002	0.000002	0.002953	0.078795	0.043592	0.104381	0.002828	0.005709	0.046748	0.007130	0.066317
ycf2	0.000001	0.000001	0.000001	0.000001	0.000001	0.011647	0.003071	0.004659	0.000002	0.000002	0.007497	0.001160	0.011710
ycf3	0.000005	0.008072	0.000005	0.007316	0.000005	0.062343	0.024671	0.025596	0.000005	0.000005	0.016464	0.000005	0.092416
ycf4	0.000003	0.000003	0.000003	0.000001	0.001684	0.047252	0.023047	0.038232	0.000004	0.000004	0.038534	0.000004	0.083096
ω													

accD	0.831572	0.831572	0.831572	0.325522	0.325522	0.179075	0.179075	0.179075	0.179075	0.179075	0.179075	0.179075	0.179075
atpA	0.082799	0.082799	0.082799	0.164067	0.164067	0.064050	0.064050	0.064050	0.064050	0.064050	0.064050	0.064050	0.064050
atpB	0.111450	0.111450	0.111450	0.078007	0.078007	0.055863	0.055863	0.055863	0.055863	0.055863	0.055863	0.055863	0.055863
atpE	0.059894	0.059894	0.059894	0.094183	0.094183	0.049331	0.049331	0.049331	0.049331	0.049331	0.049331	0.049331	0.049331
atpF	0.183988	0.183988	0.183988	0.595921	0.595921	0.150316	0.150316	0.150316	0.150316	0.150316	0.150316	0.150316	0.150316
atpH	0.000100	0.000100	0.000100	0.064919	0.064919	0.000100	0.000100	0.000100	0.000100	0.000100	0.000100	0.000100	0.000100
atpI	0.000100	0.000100	0.000100	0.027864	0.027864	0.049720	0.049720	0.049720	0.049720	0.049720	0.049720	0.049720	0.049720
ccsA	0.204737	0.204737	0.204737	0.178736	0.178736	0.265054	0.265054	0.265054	0.265054	0.265054	0.265054	0.265054	0.265054
clpP	1.396940	1.396940	1.396940	NA	NA	0.177834	0.177834	0.177834	0.177834	0.177834	0.177834	0.177834	NA
cemA	0.366243	0.366243	0.366243	0.433104	0.433104	0.264073	0.264073	0.264073	0.264073	0.264073	0.264073	0.264073	0.264073
matK	0.436284	0.436284	0.436284	0.362677	0.362677	0.319713	0.319713	0.319713	0.319713	0.319713	0.319713	0.319713	0.319713
ndhA	0.066466	0.066466	0.066466	0.208676	0.208676	0.144078	0.144078	0.144078	0.144078	0.144078	0.144078	0.144078	NA
ndhB	0.467335	0.467335	0.467335	0.113319	0.113319	0.227063	0.227063	0.227063	0.227063	0.227063	0.227063	0.227063	0.227063
ndhC	0.122280	0.122280	0.122280	0.087967	0.087967	0.093206	0.093206	0.093206	0.093206	0.093206	0.093206	0.093206	0.093206
ndhD	0.096563	0.096563	0.096563	0.173179	0.173179	0.097414	0.097414	0.097414	0.097414	0.097414	0.097414	0.097414	0.097414
ndhE	0.377302	0.377302	0.377302	0.111467	0.111467	0.082107	0.082107	0.082107	0.082107	0.082107	0.082107	0.082107	0.082107
ndhF	0.234266	0.234266	0.234266	0.181769	0.181769	0.196195	0.196195	0.196195	0.196195	0.196195	0.196195	0.196195	0.196195
ndhG	0.285374	0.285374	0.285374	0.182252	0.182252	0.228544	0.228544	0.228544	0.228544	0.228544	0.228544	0.228544	0.228544
ndhH	0.083303	0.083303	0.083303	0.052566	0.052566	0.067341	0.067341	0.067341	0.067341	0.067341	0.067341	0.067341	0.067341
ndhI	0.161166	0.161166	0.161166	0.079922	0.079922	0.106406	0.106406	0.106406	0.106406	0.106406	0.106406	0.106406	0.106406
ndhJ	0.106951	0.106951	0.106951	0.257142	0.257142	0.041309	0.041309	0.041309	0.041309	0.041309	0.041309	0.041309	0.041309
ndhK	0.157826	0.157826	0.157826	0.019716	0.019716	0.179080	0.179080	0.179080	0.179080	0.179080	0.179080	0.179080	0.179080
petA	0.105118	0.105118	0.105118	0.083816	0.083816	0.096197	0.096197	0.096197	0.096197	0.096197	0.096197	0.096197	0.096197
petB	0.019224	0.019224	0.019224	0.030394	0.030394	0.023766	0.023766	0.023766	0.023766	0.023766	0.023766	0.023766	0.023766
petD	0.241492	0.241492	0.241492	0.029899	0.029899	0.059650	0.059650	0.059650	0.059650	0.059650	0.059650	0.059650	0.059650
petG	0.259617	0.259617	0.259617	0.000100	0.000100	0.000100	0.000100	0.000100	0.000100	0.000100	0.000100	0.000100	0.000100
petL	0.089637	0.089637	0.089637	0.113840	0.113840	0.214490	0.214490	0.214490	0.214490	0.214490	0.214490	0.214490	0.214490

petN	0.000100	0.000100	0.000100	0.000100	0.000100	0.000100	0.000100	0.000100	0.000100	0.000100	0.000100	0.000100	0.000100
psaA	0.017261	0.017261	0.017261	0.018194	0.018194	0.021304	0.021304	0.021304	0.021304	0.021304	0.021304	0.021304	0.021304
psaB	0.014395	0.014395	0.014395	0.044212	0.044212	0.011398	0.011398	0.011398	0.011398	0.011398	0.011398	0.011398	0.011398
psaC	0.000100	0.000100	0.000100	0.000100	0.000100	0.000100	0.000100	0.000100	0.000100	0.000100	0.000100	0.000100	0.000100
psaI	0.552122	0.552122	0.552122	0.000100	0.000100	0.201627	0.201627	0.201627	0.201627	0.201627	0.201627	0.201627	0.201627
psaJ	0.212722	0.212722	0.212722	0.000100	0.000100	0.061470	0.061470	0.061470	0.061470	0.061470	0.061470	0.061470	0.061470
psbA	0.000100	0.000100	0.000100	0.019051	0.019051	0.022942	0.022942	0.022942	0.022942	0.022942	0.022942	0.022942	0.022942
psbB	0.042013	0.042013	0.042013	0.030433	0.030433	0.041420	0.041420	0.041420	0.041420	0.041420	0.041420	0.041420	0.041420
psbC	0.000100	0.000100	0.000100	0.078459	0.078459	0.025559	0.025559	0.025559	0.025559	0.025559	0.025559	0.025559	0.025559
psbD	0.026968	0.026968	0.026968	0.045488	0.045488	0.016529	0.016529	0.016529	0.016529	0.016529	0.016529	0.016529	0.016529
psbE	0.169252	0.169252	0.169252	0.000100	0.000100	0.000100	0.000100	0.000100	0.000100	0.000100	0.000100	0.000100	0.000100
psbF	0.000100	0.000100	0.000100	0.113037	0.113037	0.000100	0.000100	0.000100	0.000100	0.000100	0.000100	0.000100	0.000100
psbH	0.111448	0.111448	0.111448	0.102003	0.102003	0.135890	0.135890	0.135890	0.135890	0.135890	0.135890	0.135890	0.135890
psbI	0.000100	0.000100	0.000100	0.000100	0.000100	0.000100	0.000100	0.000100	0.000100	0.000100	0.000100	0.000100	0.000100
psbJ	999	999	999	19.31460	19.31460	0.173331	0.173331	0.173331	0.173331	0.173331	0.173331	0.173331	0.173331
psbK	0.340847	0.340847	0.340847	0.233349	0.233349	0.182974	0.182974	0.182974	0.182974	0.182974	0.182974	0.182974	0.182974
psbL	0.000100	0.000100	0.000100	0.000100	0.000100	0.000100	0.000100	0.000100	0.000100	0.000100	0.000100	0.000100	0.000100
psbM	0.000100	0.000100	0.000100	0.000100	0.000100	0.053087	0.053087	0.053087	0.053087	0.053087	0.053087	0.053087	0.053087
psbN	0.000100	0.000100	0.000100	0.068942	0.068942	0.028356	0.028356	0.028356	0.028356	0.028356	0.028356	0.028356	0.028356
psbT	0.000100	0.000100	0.000100	0.000100	0.000100	0.000100	0.000100	0.000100	0.000100	0.000100	0.000100	0.000100	0.000100
psbZ	0.000100	0.000100	0.000100	0.000100	0.000100	0.329409	0.329409	0.329409	0.329409	0.329409	0.329409	0.329409	0.329409
rbcL	0.040886	0.040886	0.040886	0.000100	0.000100	0.064498	0.064498	0.064498	0.064498	0.064498	0.064498	0.064498	0.064498
rpl14	0.086683	0.086683	0.086683	0.230247	0.230247	0.020292	0.020292	0.020292	0.020292	0.020292	0.020292	0.020292	0.020292
rpl16	0.113324	0.113324	0.113324	0.200162	0.200162	0.145396	0.145396	0.145396	0.145396	0.145396	0.145396	0.145396	0.145396
rpl20	0.703205	0.703205	0.703205	0.213283	0.213283	0.258350	0.258350	0.258350	0.258350	0.258350	0.258350	0.258350	0.258350
rpl22	0.418486	0.418486	0.418486	0.073771	0.073771	0.102987	0.102987	0.102987	0.102987	0.102987	0.102987	0.102987	0.102987
rpl23	0.000100	0.000100	0.000100	0.187497	0.187497	0.232344	0.232344	0.232344	0.232344	0.232344	0.232344	0.232344	0.232344

rpl2	0.136778	0.136778	0.136778	0.171973	0.171973	0.130217	0.130217	0.130217	0.130217	0.130217	0.130217	0.130217	0.130217
rpl32	0.050644	0.050644	0.050644	2.813520	2.813520	0.080142	0.080142	0.080142	0.080142	0.080142	0.080142	0.080142	NA
rpl33	0.056248	0.056248	0.056248	0.426540	0.426540	0.202943	0.202943	0.202943	0.202943	0.202943	0.202943	0.202943	0.202943
rpl36	0.000100	0.000100	0.000100	0.000100	0.000100	0.015609	0.015609	0.015609	0.015609	0.015609	0.015609	0.015609	0.015609
rpoA	0.240341	0.240341	0.240341	0.279235	0.279235	0.218103	0.218103	0.218103	0.218103	0.218103	0.218103	0.218103	0.218103
rpoB	0.173936	0.173936	0.173936	0.193121	0.193121	0.097981	0.097981	0.097981	0.097981	0.097981	0.097981	0.097981	0.097981
rpoC1	0.271706	0.271706	0.271706	0.211632	0.211632	0.128190	0.128190	0.128190	0.128190	0.128190	0.128190	0.128190	0.128190
rpoC2	0.233307	0.233307	0.233307	0.335061	0.335061	0.232409	0.232409	0.232409	0.232409	0.232409	0.232409	0.232409	0.232409
rps11	0.595111	0.595111	0.595111	0.596974	0.596974	0.097880	0.097880	0.097880	0.097880	0.097880	0.097880	0.097880	0.097880
rps12	999	999	999	0.883367	0.883367	0.378068	0.378068	0.378068	0.378068	0.378068	0.378068	0.378068	0.378068
rps14	1.239120	1.239120	1.239120	0.322319	0.322319	0.160744	0.160744	0.160744	0.160744	0.160744	0.160744	0.160744	0.160744
rps15	0.237477	0.237477	0.237477	0.318774	0.318774	0.280507	0.280507	0.280507	0.280507	0.280507	0.280507	0.280507	0.280507
rps18	1.515030	1.515030	1.515030	2.003190	2.003190	0.000100	0.000100	0.000100	0.000100	0.000100	0.000100	0.000100	0.000100
rps19	2.400060	2.400060	2.400060	0.281326	0.281326	0.000100	0.000100	0.000100	0.000100	0.000100	0.000100	0.000100	0.000100
rps2	0.309593	0.309593	0.309593	0.310746	0.310746	0.222091	0.222091	0.222091	0.222091	0.222091	0.222091	0.222091	0.222091
rps3	0.215400	0.215400	0.215400	0.727419	0.727419	0.121757	0.121757	0.121757	0.121757	0.121757	0.121757	0.121757	0.121757
rps4	NA	NA	NA	0.666998	0.666998	0.100938	0.100938	0.100938	0.100938	0.100938	0.100938	0.100938	0.100938
rps7	2.222420	2.222420	2.222420	0.926943	0.926943	0.104884	0.104884	0.104884	0.104884	0.104884	0.104884	0.104884	0.104884
rps8	0.042713	0.042713	0.042713	0.211304	0.211304	0.157008	0.157008	0.157008	0.157008	0.157008	0.157008	0.157008	0.157008
rps16	1.375870	1.375870	1.375870	NA	NA	0.183226	0.183226	0.183226	0.183226	0.183226	0.183226	0.183226	NA
ycf1	0.508339	0.508339	0.508339	0.716104	0.716104	0.394305	0.394305	0.394305	0.394305	0.394305	0.394305	0.394305	0.394305
ycf2	1.103700	1.103700	1.103700	0.934427	0.934427	0.478369	0.478369	0.478369	0.478369	0.478369	0.478369	0.478369	0.478369
ycf3	0.039409	0.039409	0.039409	0.071999	0.071999	0.030400	0.030400	0.030400	0.030400	0.030400	0.030400	0.030400	0.030400
ycf4	0.381179	0.381179	0.381179	1.153970	1.153970	0.092963	0.092963	0.092963	0.092963	0.092963	0.092963	0.092963	0.092963

Table S6. Summary of the results of the m0 and m2 models analyzed in the study. The d_N, d_S and ω median values are provided.

	m0				m2											
Gene	d_{N0}	d_{S0}	ω_0	$\ln L_0$	d_{N2}	d_{NC}	d_{NM}	d_{S2}	d_{SC}	d_{SM}	ω_2	ω_C	ω_M	$\ln L_2$	$2\Delta \ln L_{2\text{-}0}$	*P*-value
accD	0.0007	0.0025	0.2962	-3183.55	0.004625	0.001064	0.000001	0.025828	0.001280	0.000003	0.179075	0.831572	0.325522	-3172.48	22.15	0.000003
atpA	0.0002	0.0022	0.0861	-3443.96	0.001307	0	0.000459	0.020396	0.000004	0.002797	0.064050	0.082799	0.164067	-3440.33	7.25	0.01
atpB	0.0005	0.0069	0.07	-3273.54	0.001181	0	0.000435	0.021128	0.000004	0.005571	0.055863	0.111450	0.078007	-3272.33	2.42	0.12
atpE	0.0006	0.0091	0.0625	-910.89	0.000942	0	0	0.019096	0.000005	0.000004	0.049331	0.059894	0.094183	-910.38	1.01	0.31
atpF	0	0	0.2054	-1227.00	0.002130	0.000001	0.000831	0.014165	0.000004	0.001395	0.150316	0.183988	0.595921	-1223.98	6.03	0.01
atpH	0	0	0.0092	-469.67	0.000002	0	0	0.017217	0.000005	0.000004	0.000100	0.000100	0.064919	-467.81	3.72	0.05
atpI	0	0	0.0399	-1479.46	0.001170	0	0	0.023520	0.000005	0.000005	0.049720	0.000100	0.027864	-1478.11	2.69	0.10
ccsA	0.0035	0.0143	0.2421	-3094.83	0.018126	0.000001	0.001461	0.068386	0.000004	0.008174	0.265054	0.204737	0.178736	-3093.87	1.91	0.17
clpP	0.0016	0.0023	0.6943	-1644.34	0.001593	0.000001	NA	0.008959	0.000001	NA	0.177834	1.396940	NA	-1630.74	27.20	0.00
cemA	0.001	0.0032	0.3078	-1968.18	0.002677	0.000001	0.001143	0.010137	0.000003	0.002638	0.264073	0.366243	0.433104	-1967.28	1.81	0.18
matK	0.002	0.0058	0.3395	-4969.66	0.014112	0.000001	0.001528	0.044139	0.000002	0.004212	0.319713	0.436284	0.362677	-4968.99	1.34	0.25
ndhA	0.0009	0.00665	0.1384	-2618.90	0.006467	0	0.000731	0.044888	0.000004	0.003504	0.144078	0.066466	0.208676	-2616.35	5.09	0.02
ndhB	0	0	0.2279	-2434.02	0.000376	0.000001	0	0.001657	0.000002	0.000004	0.227063	0.467335	0.113319	-2432.48	3.07	0.08
ndhC	0	0	0.0949	-806.30	0.001304	0	0	0.013985	0.000004	0.000004	0.093206	0.122280	0.087967	-806.26	0.09	0.77
ndhD	0.0007	0.0066	0.1071	-4175.40	0.006755	0	0.000993	0.069340	0.000004	0.005734	0.097414	0.096563	0.173179	-4173.48	3.84	0.05
ndhE	0.0012	0.0118	0.1053	-762.70	0.003358	0.000001	0.000630	0.040892	0.000003	0.005655	0.082107	0.377302	0.111467	-761.13	3.15	0.08
ndhF	0.0017	0.0083	0.1987	-7227.14	0.014208	0.000001	0.000786	0.072416	0.000003	0.004322	0.196195	0.234266	0.181769	-7226.67	0.93	0.33
ndhG	0.0011	0.0047	0.2253	-1436.25	0.010008	0.000001	0.001369	0.043788	0.000003	0.007511	0.228544	0.285374	0.182252	-1435.97	0.56	0.45
ndhH	0.0004	0.0064	0.0672	-2849.31	0.002543	0	0.000344	0.037768	0.000004	0.006535	0.067341	0.083303	0.052566	-2848.93	0.75	0.39
ndhI	0.0007	0.0068	0.1048	-1357.44	0.003274	0.000001	0.000285	0.030768	0.000004	0.003565	0.106406	0.161166	0.079922	-1357.01	0.88	0.35
ndhJ	0.0006	0.0079	0.0763	-1085.04	0.001390	0	0.000666	0.033638	0.000004	0.002588	0.041309	0.106951	0.257142	-1080.71	8.66	0.003251
ndhK	0.0006	0.0044	0.1466	-1537.41	0.001852	0.000001	0	0.010342	0.000004	0.000005	0.179080	0.157826	0.019716	-1533.78	7.26	0.01
petA	0.0003	0.0035	0.095	-2431.98	0.003024	0	0.000150	0.031435	0.000004	0.001790	0.096197	0.105118	0.083816	-2431.88	0.19	0.66

petB	0	0	0.0244	-1376.13	0.000745	0	0	0.031333	0.000005	0.000005	0.023766	0.019224	0.030394	-1376.06	0.14	0.71
petD	0.0005	0.0072	0.0656	-1189.47	0.001739	0.000001	0	0.029146	0.000003	0.000005	0.059650	0.241492	0.029899	-1186.82	5.31	0.02
petG	0	0	0.0371	-216.78	0	0.000001	0	0.000004	0.000003	0.000004	0.000100	0.259617	0.000100	-215.04	3.49	0.06
petL	0	0	0.1551	-234.52	0.005838	0	0	0.027218	0.000004	0.000004	0.214490	0.089637	0.113840	-234.21	0.62	0.43
petN	0	0	0.0001	-122.90	0	0	0	0.000005	0.000005	0.000005	0.000100	0.000100	0.000100	-122.90	0.00	1.00
psaA	0.0001	0.0031	0.02	-4566.49	0.000488	0	0.000062	0.022910	0.000005	0.003393	0.021304	0.017261	0.018194	-4566.42	0.13	0.72
psaB	0.0001	0.0036	0.0182	-4310.71	0.000254	0	0.000074	0.022284	0.000005	0.001682	0.011398	0.014395	0.044212	-4308.02	5.39	0.02
psaC	0	0.04	0.0001	-520.82	0.000005	0	0	0.049443	0.000006	0.000006	0.000100	0.000100	0.000100	-520.82	0.00	1.00
psaI	0	0	0.2321	-224.75	0.000001	0.000001	0	0.000003	0.000002	0.000005	0.201627	0.552122	0.000100	-223.56	2.39	0.12
psaJ	0	0	0.0821	-289.43	0.000654	0.000001	0.000001	0.010640	0.000003	0.012216	0.061470	0.212722	0.000100	-288.34	2.19	0.14
psbA	0.0001	0.0038	0.0198	-1930.93	0.000533	0	0.000036	0.023205	0.000005	0.001875	0.022942	0.000100	0.019051	-1930.25	1.36	0.24
psbB	0.0001	0.0024	0.0389	-3263.17	0.001157	0	0	0.027933	0.000004	0.000005	0.041420	0.042013	0.030433	-3262.98	0.37	0.54
psbC	0.0001	0.0025	0.0331	-2814.42	0.000495	0	0.000088	0.019359	0.000005	0.001122	0.025559	0.000100	0.078459	-2810.83	7.19	0.01
psbD	0	0	0.0229	-1917.41	0.000154	0	0.000073	0.009319	0.000004	0.001591	0.016529	0.026968	0.045488	-1916.88	1.06	0.30
psbE	0	0	0.018	-421.10	0	0.000001	0	0.000005	0.000003	0.000005	0.000100	0.169252	0.000100	-419.06	4.08	0.04
psbF	0	0	0.0301	-214.70	0	0	0.001450	0.000005	0.000005	0.012828	0.000100	0.000100	0.113037	-213.49	2.42	0.12
psbH	0	0	0.1253	-574.84	0.002840	0	0.000744	0.020894	0.000004	0.007290	0.135890	0.111448	0.102003	-574.75	0.18	0.67
psbI	0	0	0.0001	-199.99	0.000002	0	0	0.013746	0.000004	0.000004	0.000100	0.000100	0.000100	-199.99	0.00	1.00
psbJ	0	0	0.2333	-203.99	0.001931	0.000002	0.000002	0.011137	0	0	0.173331	999	19.314600	-202.95	2.08	0.15
psbK	0.0029	0.0136	0.2118	-385.51	0.002648	0.000001	0.000001	0.014468	0.000003	0.000003	0.182974	0.340847	0.233349	-385.35	0.31	0.57
psbL	0	0	0.0001	-181.65	0.000002	0	0	0.017260	0.000006	0.000006	0.000100	0.000100	0.000100	-181.65	0.00	1.00
psbM	0	0	0.0301	-176.75	0	0	0	0.000004	0.000005	0.000005	0.053087	0.000100	0.000100	-176.22	1.05	0.31
psbN	0	0	0.0362	-271.39	0.000688	0	0	0.024243	0.000005	0.000004	0.028356	0.000100	0.068942	-271.03	0.72	0.40
psbT	0	0	0.0001	-182.72	0	0	0	0.000005	0.000005	0.000005	0.000100	0.000100	0.000100	-182.72	0.00	1.00
psbZ	0	0	0.1875	-299.00	0.000036	0	0	0.000108	0.000005	0.000005	0.329409	0.000100	0.000100	-297.33	3.34	0.07
rbcL	0.0002	0.0051	0.0479	-2907.48	0.001366	0	0	0.021176	0.000005	0.001457	0.064498	0.040886	0.000100	-2901.93	11.09	0.000868
rpl14	0	0	0.0865	-814.04	0.000304	0	0.000001	0.014984	0.000005	0.000003	0.020292	0.086683	0.230247	-808.62	10.84	0.000995

rpl16	0.0011	0.0072	0.1508	-1008.91	0.002145	0	0.000648	0.014751	0.000004	0.003236	0.145396	0.113324	0.200162	-1008.61	0.60	0.44
rpl20	0	0	0.3025	-978.82	0.003607	0.000001	0.000001	0.013962	0.000002	0.000003	0.258350	0.703205	0.213283	-976.76	4.11	0.04
rpl22	0	0	0.1331	-1350.77	0.004203	0.000001	0	0.040814	0.000003	0.000005	0.102987	0.418486	0.073771	-1345.61	10.33	0.001309
rpl23	0	0	0.1956	-483.41	0.000001	0	0.002011	0.000003	0.000006	0.010723	0.232344	0.000100	0.187497	-483.39	0.04	0.85
rpl2	0	0	0.1487	-1499.84	0.000263	0.000001	0.000001	0.002019	0.000004	0.000004	0.130217	0.136778	0.171973	-1499.67	0.34	0.56
rpl32	0.002	0.0092	0.2182	-395.56	0.005476	0	0.017960	0.068327	0.000005	0.006384	0.080142	0.050644	2.813520	-390.28	10.56	0.001156
rpl33	0	0	0.1725	-485.51	0.005801	0	0.000001	0.028583	0.000005	0.000002	0.202943	0.056248	0.426540	-483.88	3.27	0.07
rpl36	0	0	0.0106	-270.54	0.000267	0	0	0.017110	0.000005	0.000005	0.015609	0.000100	0.000100	-270.17	0.75	0.39
rpoA	0.0006	0.0027	0.2344	-2711.03	0.007637	0.000001	0.000349	0.035013	0.000003	0.001247	0.218103	0.240341	0.279235	-2710.73	0.61	0.43
rpoB	0.0004	0.0031	0.1276	-7589.91	0.002641	0.000001	0.000326	0.026951	0.000004	0.001689	0.097981	0.173936	0.193121	-7583.85	12.13	0.000496
rpoC1	0.0005	0.0028	0.1672	-4821.76	0.002923	0.000001	0.000415	0.022802	0.000003	0.001960	0.128190	0.271706	0.211632	-4817.66	8.20	0.004196
rpoC2	0.0006	0.0024	0.2535	-11172.54	0.005767	0.000001	0.000174	0.024812	0.000003	0.000518	0.232409	0.233307	0.335061	-11169.65	5.78	0.02
rps11	0.0014	0.0054	0.2656	-1170.08	0.002010	0.000001	0.002100	0.020530	0.000002	0.003518	0.097880	0.595111	0.596974	-1160.96	18.24	0.000020
rps12	0	0	0.5826	-697.18	0.001722	0.000002	0.000001	0.004555	0	0.000001	0.378068	999	0.883367	-696.16	2.04	0.15
rps14	0	0	0.2672	-761.86	0.000381	0.000001	0.001150	0.002369	0.000001	0.003566	0.160744	1.239120	0.322319	-760.34	3.04	0.08
rps15	0.0029	0.0101	0.2838	-766.45	0.005666	0.000001	0.004671	0.020199	0.000003	0.014651	0.280507	0.237477	0.318774	-766.35	0.20	0.65
rps18	0.0008	0.0013	0.6016	-269.93	0.000003	0.000001	0.011500	0.023526	0.000001	0.005741	0.000100	1.515030	2.003190	-263.01	13.86	0.000197
rps19	0	0	0.0237	-403.69	0	0.000002	0.000001	0.000007	0.000001	0.000003	0.000100	2.400060	0.281326	-401.16	5.06	0.02
rps2	0.0009	0.0035	0.2536	-1619.19	0.002787	0.000001	0.000497	0.012551	0.000003	0.001598	0.222091	0.309593	0.310746	-1618.80	0.78	0.38
rps3	0.001	0.0044	0.2226	-1796.75	0.004952	0.000001	0.000001	0.040672	0.000004	0.000002	0.121757	0.215400	0.727419	-1784.88	23.75	0.000001
rps4	0.0015	0.00715	0.2099	-1256.43	0.001136	NA	0.001642	0.011252	NA	0.002462	0.100938	NA	0.666998	-1247.65	17.56	0.000028
rps7	0	0	0.5012	-943.63	0.000803	0.000002	0.001078	0.007652	0.000001	0.001163	0.104884	2.222420	0.926943	-936.16	14.95	0.000110
rps8	0	0	0.1511	-889.79	0.003495	0	0.000693	0.022259	0.000005	0.003276	0.157008	0.042713	0.211304	-888.68	2.23	0.14
rps16	0	0.00035	0.4929	-520.66	0.000001	0.000001	NA	0.000004	0.000001	NA	0.183226	1.375870	NA	-516.74	7.85	0.01
ycf1	0.0024	0.0052	0.4682	-5225.35	0.017811	0.000001	0.001058	0.045170	0.000002	0.001478	0.394305	0.508339	0.716104	-5221.31	8.08	0.004467
ycf2	0	0	0.756	-10182.30	0.001849	0.000001	0.000001	0.003865	0.000001	0.000001	0.478369	1.103700	0.934427	-10174.40	15.81	0.000070
ycf3	0.0003	0.008	0.0402	-999.54	0.000626	0	0.000264	0.020568	0.000005	0.003661	0.030400	0.039409	0.071999	-999.04	1.00	0.32

ycf4	0	0	0.2783	-1626.02	0.002848	0.000001	0.000972	0.030640	0.000003	0.000843	0.092963	0.381179	1.153970	-1606.80	38.43	0.00

Table S7. The list of amino acid sites for the eleven genes under positive selection.

psbJ		
Species	a.a.	Type
C. falcata	FV	A
C. testiculata	FV	A
C. orthoceras	FV	A
M. apetalus	LV	C
M. minimus	LV	C
R. membranaceus	LI	B
rps18		
Species	a.a.	Type
C. falcata	FRSRRPIGQYYKLISR	B
C. testiculata	FQSRRPIGQYYKLISR	A
C. orthoceras	FRSRRPIGQYYKLISR	B
M. apetalus	SQPCCTMRRYSNFTNQ	C
M. minimus	SQPCRTMRLYSNFTNQ	D
R. membranaceus	SRSRRPIGRDYNLISR	E
rpl32		
Species	a.a.	Type
C. falcata	APSRRCWKYAIKSS	A
C. testiculata	APSRRCWKYAIKSS	A
C. orthoceras	APSRRCWKYAIKSS	A
M. apetalus	TSFHPLRNYTLRSI	C
M. minimus	TSFHRLRNYALRYN	D
R. membranaceus	APSRRFWNSAIKSS	B
ycf4		
Species	a.a.	Type
C. falcata	HEIETFGMFTIQEPSMVAGLSCLCTLSNERTKMQFLRRDCINEMIRHAEEMQRAEHRY	A
C. testiculata	HEIETFGMFTIQEPSMVAGLSCLCTLSNERTKMQFLRRDCINEMIRHAEEMQRAEHRY	A
C. orthoceras	HEIETFGMFTIQEPSMVAGLSCLCTLSNERTKMQFLRRDCINEMIRHAEEMQRAEHRY	A
M. apetalus	TKMDSCFMLITPEPPVISSITYLYAWGRDTTELYSELIASMKDLLLQDEQIEMIQSDP	D
M. minimus	TKMDSCFMLITPEPPVISSITYFYAWGRDTTELYSELIASMKDLLLQDEQIEMIQSDP	C
R. membranaceus	NEIETFCTFTIQQLPMIAGLSSLWTSSRDRKKICFLMRDSIKEMIRQAQEIQKAEYRV	B
clpP		
Species	a.a.	Type
C. falcata	RCLDEETIIYQIFVKRIMLAGIGCMFNDKMEFMYGGMSATEATFKVIAHGLTNSKIGDLF ICSYLNDGIFRDLIIAKSYEISLNEYQIKYIDLIDGLTGVI	B
C. testiculata	RCLDEETIIYQIFVKRIMLAGIGCMFNDKMEFMYGGMSATEATFKVIAHGLTNSKIGDLF ICSYLNDGIFRDLIIAKSYEISLNEYQIKYIDLIDGLTGVI	B
C. orthoceras	RCLDEETIIYQIFVKRIMLAGIGCMFNDKMEFMYGGMSATEATFKVIAHGLTNSKIGDLF ICSYLNDGIFRDLIIAKSYEISLNEYQIKYIDLIDGLTGVI	B
M. apetalus	NA	
M. minimus	NA	
R. membranaceus	KPSEDAVVVHELLLQGVEISNLIGLMSEERDLLFWPVATFPPHMLAMGVTIPHAWVASF YEATFVAEELLKETLVVNPLWVEMEVFTQAHVLVVENTGDFT	A
rps7		
Species	a.a.	Type
C. falcata	SKVGALKTSEIRALSHTNVPKRASKPFGVAI	D

C. testiculata	SKVGALKTSEIRALSHTNVPKRASKPFGVAI	D
C. orthoceras	SKVGALKTSEIRALSHTNVPKRASKPFGVAI	D
M. apetalus	AKLVALNKPQVRNIGQIEISRKVARLLDISF	A
M. minimus	SKLVALNKPQVRNIGQIEISRKVARLLDISF	C
R. membranaceus	SELVSIKTSQIGDIGHIEISTKASRPFDIAF	B
rps12		
Species	a.a.	Type
C. falcata	INTCPATITAIIAAGDK	A
C. testiculata	INTCPATITAIIAAGDK	A
C. orthoceras	INTCPATITAIIAAGDK	A
M. apetalus	SKKTRHTITGVVATAGK	C
M. minimus	SKKTRHTITGVVATAGK	C
R. membranaceus	INTSPAVYHAIIGAGDQ	B
rps14		
Species	a.a.	Type
C. falcata	RHSQIQVSLSESKAKLQSNASH	B
C. testiculata	RHSQIQVSLSESKAKLQSNASH	B
C. orthoceras	RHSQIQVSLSESKAKLQSNASH	B
M. apetalus	RRIELKIANLDTMKQMMQDSLQ	C
M. minimus	RRIELKIANLDTMKQMMQDSLQ	C
R. membranaceus	KHSEIKVSLSEWHGKLQSNASH	A
rps16		
Species	a.a.	Type
C. falcata	FNAIQGGLLRFNFKMIYEN	A
C. testiculata	FNAIQGGLLRFNFKMIYEN	A
C. orthoceras	FNAIQGGLLRFNFKMIYEN	A
M. apetalus	NA	
M. minimus	NA	
R. membranaceus	LKGVREVPIQPALETVDKK	B
rps19		
Species	a.a.	Type
C. falcata	HTDGMVHKLEFSPTLNREHSRN	A
C. testiculata	HTDGMVHKLEFSPTLNREHSRN	A
C. orthoceras	HTDGMVHKLEFSPTLNREHSRN	A
M. apetalus	NYNRWYRQIRICTYFAPGTCEK	C
M. minimus	NYNRWYRQIRICTYFAPGTCEK	C
R. membranaceus	HTDRMVHKLEFAPTLNRGHARN	B
ycf2		
Species	a.a.	Type
C. falcata	QDHRPFKSNKLANQSANQYFSPLFLSSLLSLPSFVLIHIQCPLESIQLSTSPWEKRRWLNH PWRYKDRIRKRHEELYLDSHRLAGIAHPYIPKCLKQCKMYTFQRRWWFTCKTSTLTPRS ISQKWHRNISIPLGWIRSCLLSRIHDPIWPRDYLRTHLFVRREIRYTRKDKFVLAGGFVIIV DRSTRLYAIQVAEWDAQVSTTMTHFDSRIASVFVKLPFPSTVDETKKVSSDESTDDIIIDL ERMTEVIMNTPRIDRIADDLSKNLCSLNNRNDAHRWAASSRNQEVYGTYVFLPYNVTL TSYVFDEEEEDFNHWGIYWRSHEFGQFVKPSGLEFDRSRKKSLKLIKARQYRNFYLRV	C

C. testiculata	QDHRPFKSNKLANQSANQYFSPLFLSSLLSLPSFVLIHIQCPLESIQLSTSPWEKRRWLNH<br>PWRYKDRIRKRHEELYLDSHRLAGIAHPYIPKCLKQCKMYTFQRRWWFTCKTSTLTPRS<br>ISQKWHRNISIPLGWIRSCLLSRIHDPIWPRDYLRTHLFVRREIRYTRKDKFVLAGGFVIIV<br>DRSTRLYAIQVAEWDAQVSTTMTHFDSRIASVFVKLPFPSTVDETKKVSSDESTDDIIIDL<br>ERMTEVIMNTPRIDRIADDLSKNLCSLNNRNDAHRWAASSRNQEVYGTYVFLPYNVTL<br>TSYVFDEEEEDFNHWGIYWRSHEFGQFVKPSGLEFDRSRKKSLKLIKARQYRNFYLRV	C
C. orthoceras	QDHRPFKSNKLANQSANQYFSPLFLSSLLSLPSFVLIHIQCPLESIQLSTSPWEKRRWLNH<br>PWRYKDRIRKRHEELYLDSHRLAGIAHPYIPKCLKQCKMYTFQRRWWFTCKTSTLTPRS<br>ISQKWHRNISIPLGWIRSCLLSRIHDPIWPRDYLRTHLFVRREIRYTRKDKFVLAGGFVIIV<br>DRSTRLYAIQVAEWDAQVSTTMTHFDSRIASVFVKLPFPSTVDETKKVSSDESTDDIIIDL<br>ERMTEVIMNTPRIDRIADDLSKNLCSLNNRNDAHRWAASSRNQEVYGTYVFLPYNVTL<br>TSYVFDEEEEDFNHWGIYWRSHEFGQFVKPSGLEFDRSRKKSLKLIKARQYRNFYLRV	C
M. apetalus	EEYGQLKSNNTVDWLQDRELPSLLFFVFLLPFFLLFTRVRGPVQFLKLPIGARDNCSLPK<br>DRGISRSKTDNIAADSSIEPRCRVERVHLSKRKRSLCWELQSLSKKWYCIYNKWVFILIR<br>LGLQFAVQKKMKQWRVDTCFIIPLQDQLSPEACLHRDVLAGSKSQDLETDSLVSEWRS<br>FRLIHQLRRIKASNLGDLEREVCNRMIPLGLTSVRASLTLASANPYTERIFLDILDETPMW<br>SFSVKLKATLASNASELGELVNNRCKDFLCWSSQKGAKQPVVNIKREAGSWASFVPPS<br>NMALNYDLVYDDGAALTNWRLSGKYPLSRRSLQTFGLQSKGPTEKVLELMNAQEHHK<br>SYWKG	A
M. minimus	EEYGQLKSNNTVDWLQDRELPSLLFFVFLLPFFLLFTRVRGPVQFLKLPIGARDNCSLPK<br>DRGISRSKTDNIAADSSIEPRCRVERVHLSKRKRSLCWELQSLSKKWYCIYNKWVFILIR<br>LGLQFAVQKKMKQWRVDTCFIIPLQDQLSPEACLHRDVLAGSKSQDLETDSLVSEWRS<br>FRLIHQLRRIKASNLGDLEREVCNRMIPLGLTSVRASLTLASANPYTERIFLDILDETPMW<br>SFSVKLKATLASNASELGELVNNRCKDFLCWSSQKGAKQPVVNIKREAGSWASFVPPS<br>NMALNYDLVYDDGAALTNWRLSGKYPLSRRSLQTFGLQSKGPTEKVLELMNAQEHHK<br>SYWKG	A
R. membranaceus	QDHIQLTNKKLVNQSEDQNFSSFFLSSLSSLLSFVIIHIQCQLESIQWSTSPWENRRWLNH<br>PWRYKAKIRKIHEESSIDSHRLAGIVNPYMPECLKKCKMYTFQRKLWLACKNFTLTPISI<br>SQKWHGDISIPQLWIPSFLLSTIRHPIWAREYFRTHLFVRSEIRYTRKNKFGSERGSVILVD<br>RSTGINDIKVADSDEELSATITHLDSTIASASVTVTFPNKLDDKKLDSDIDSDDISIDLELM<br>TALTMYTPKIDRIVNNLCQNSCYLTNRKDSQRSVANIKKKEGYGTYFVPQYKVTFNSY<br>VFYEEGEDFNHCGIYWKYHKSGRFLKQFVFQSKGSTNTSIKFINTQEHHNSSLRV	B

Table S8. The results of different heterogeneous rate model tests for the 11 gene under positive selection. ER, equal-rates likelihood model; SYM, symmetrical likelihood model; ARD, all-rates-different likelihood model.

Genus	Gene	Age	lnL_{ER}	lnL_{SYM}	lnL_{ARD}	$2\Delta lnL_{ARD-ER}$	*P* value
Ceratocephala and *Myosurus*	*psbJ*	3.3	-3.62	-3.15	-2.96	1.32	0.25
		13.9	-3.30	-3.26	-3.25	0.10	0.76
		17	-3.30	-3.30	-3.28	0.04	0.84
		23.03	-3.30	-3.30	-3.29	0.01	0.93
		34	-3.42	-3.41	-3.40	0.02	0.89
	rps18	3.3	-9.66	-6.94	-6.85	5.62	0.02
		13.9	-9.66	-6.92	-6.53	6.25	0.01
		17	-9.66	-6.90	-6.57	6.17	0.01
		23.03	-9.66	-6.91	-6.59	6.13	0.01
		34	-9.66	-7.01	-7.12	5.08	0.02
Ceratocephala	*clpP*	3.3	-1.39	-1.39	-1.39	0.00	1.00
		13.9	-1.39	-1.39	-1.39	0.00	1.00
		17	-1.39	-1.39	-1.39	0.00	1.00
		23.03	-1.39	-1.39	-1.39	0.00	1.00
		34	-1.39	-1.39	-1.39	0.00	1.00
	rps7	3.3	-6.40	-4.81	-4.32	4.16	0.04
		13.9	-6.40	-4.83	-4.62	3.58	0.06
		17	-6.40	-4.85	-4.65	3.51	0.06
		23.03	-6.40	-4.85	-4.67	3.46	0.06
		34	-5.55	49.83	-4.78	1.54	0.21
	rps12	3.3	-3.62	-3.15	-2.96	1.32	0.25
		13.9	-3.30	-3.26	-3.25	0.10	0.76
		17	-3.30	-3.30	-3.28	0.04	0.84
		23.03	-3.30	-3.30	-3.29	0.01	0.93
		34	-3.42	-3.41	-3.40	0.02	0.89
	rps14	3.3	-3.62	-3.15	-2.95	1.34	0.25
		13.9	-3.30	-3.26	-3.25	0.10	0.76
		17	-3.30	-3.30	-3.28	0.04	0.84
		23.03	-3.30	-3.30	-3.29	0.01	0.93
		34	-3.42	-3.41	-3.39	0.05	0.83
	rps16	3.3	-1.39	-1.39	-1.39	0.00	1.00
		13.9	-1.39	-1.39	-1.39	0.00	1.00
		17	-1.39	-1.39	-1.39	0.00	1.00
		23.03	-1.39	-1.39	-1.39	0.00	1.00
		34	-1.39	-1.39	-1.39	0.00	1.00
	rps19	3.3	-3.62	-3.15	-2.96	1.32	0.25
		13.9	-3.30	-3.26	-3.25	0.10	0.76
		17	-3.30	-3.30	-3.28	0.04	0.84
		23.03	-3.30	-3.30	-3.29	0.01	0.93
		34	-3.42	-3.41	-3.40	0.02	0.89
	ycf2	3.3	-3.62	-3.15	-2.95	1.34	0.25
		13.9	-3.30	-3.26	-3.25	0.10	0.76
		17	-3.30	-3.30	-3.28	0.04	0.84
		23.03	-3.30	-3.30	-3.29	0.01	0.93
		34	-3.42	-3.41	-3.39	0.05	0.83
Myosurus	*rpl32*	3.3	-6.40	-4.81	-4.32	4.16	0.04
		13.9	-6.40	-4.83	-4.62	3.58	0.06
		17	-6.40	-4.84	-4.65	3.51	0.06
		23.03	-6.40	-4.85	-4.67	3.46	0.06
		34	-5.55	-4.94	-4.76	1.56	0.21
	ycf4	3.3	-6.40	-4.81	-4.32	4.16	0.04

13.9	-6.40	-4.83	-4.62	3.58	0.06
17	-6.40	-4.84	-4.65	3.51	0.06
23.03	-6.40	-4.85	-4.67	3.46	0.06
34	-5.55	-4.94	-4.76	1.56	0.21
